# A Robust Framework for Generating Adsorption Isotherms to Screen Materials for Carbon Capture

**DOI:** 10.1021/acs.iecr.3c01358

**Published:** 2023-06-20

**Authors:** Elias Moubarak, Seyed Mohamad Moosavi, Charithea Charalambous, Susana Garcia, Berend Smit

**Affiliations:** † Laboratory of Molecular Simulation (LSMO), Institut des Sciences et Ingénierie Chimiques, École Polytechnique Fédérale de Lausanne (EPFL), Rue de l’Industrie 17, CH-1951 Sion, Valais, Switzerland; ‡ Department of Chemical Engineering & Applied Chemistry, University of Toronto, Toronto, Ontario M5S 3E5, Canada; § The Research Centre for Carbon Solutions (RCCS), School of Engineering and Physical Sciences, Heriot-Watt University, EH14 4AS Edinburgh, United Kingdom

## Abstract

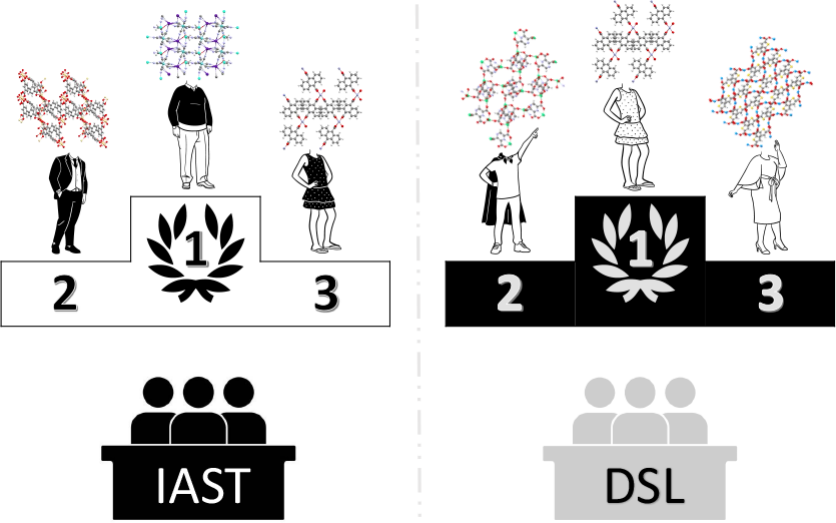

To rank the performance of materials for a given carbon capture process, we rely on pure component isotherms from which we predict the mixture isotherms. For screening a large number of materials, we also increasingly rely on isotherms predicted from molecular simulations. In particular, for such screening studies, it is important that the procedures to generate the data are accurate, reliable, and robust. In this work, we develop an efficient and automated workflow for a meticulous sampling of pure component isotherms. The workflow was tested on a set of metal–organic frameworks (MOFs) and proved to be reliable given different guest molecules. We show that the coupling of our workflow with the Clausius–Clapeyron relation saves CPU time, yet enables us to accurately predict pure component isotherms at the temperatures of interest, starting from a reference isotherm at a given temperature. We also show that one can accurately predict the CO_2_ and N_2_ mixture isotherms using ideal adsorbed solution theory (IAST). In particular, we show that IAST is a more reliable numerical tool to predict binary adsorption uptakes for a range of pressures, temperatures, and compositions, as it does not rely on the fitting of experimental data, which typically needs to be done with analytical models such as dual-site Langmuir (DSL). This makes IAST a more suitable and general technique to bridge the gap between adsorption (raw) data and process modeling. To demonstrate this point, we show that the ranking of materials, for a standard three-step temperature swing adsorption (TSA) process, can be significantly different depending on the thermodynamic method used to predict binary adsorption data. We show that, for the design of processes that capture CO_2_ from low concentration (0.4%) streams, the commonly used methodology to predict mixture isotherms incorrectly assigns up to 33% of the materials as top-performing.

## Introduction

Carbon dioxide, considered one of the main greenhouse gases, has a major impact on climate change. Its atmospheric concentration is already above 400 ppm and is predicted to hit alarming levels in the 2050s^1^ if the current situation is not addressed properly. Emission sources are numerous and contribute differently to the problem. They range from deforestation and cement manufacturing to the burning of fossil fuels such as natural gas, petroleum, and coal. Consequently, sectors relying heavily on fossil fuels, such as the power generation, industrial, and transportation sectors, are the main sources of CO_2_ emissions worldwide.^2^ Current carbon capture and storage technologies have the potential to mitigate climate change. However, they are energy intensive.^3^ Chemical absorption using amine solutions to recover CO_2_ from flue gas streams is well-developed and deployed on a commercial scale. Also, polymeric membranes are being used commercially to separate CO_2_ from syngas.^3−5^ Reaching the full potential within the time limit imposed by the fact that CO_2_ emissions are still increasing has motivated research to capture CO_2_ directly from atmospheric air.^6^ Adsorption-based technologies are promising candidates that address these challenges: to efficiently capture CO_2_ from a wide range of sources.

To accelerate the pace of development and provide cost-effective solutions for carbon capture applications, the materials and process engineering research communities have shifted from assessing a limited number of structures at a time toward screening entire databases for evaluating the performance of hundreds to millions of solid sorbents.^7−15^ In some of these studies,^14,15^ simple metrics such as selectivity were used to evaluate the performance of the materials. In a more recent study,^16^ a vacuum swing adsorption process was developed and materials were ranked on the basis of process indicators such as purity, recovery, parasitic energy, and productivity. These screening studies consider mainly binary mixtures of CO_2_ and N_2_ in the gas stream. Therefore, the accurate prediction of binary adsorption data plays a key role in evaluating the performance of materials in carbon capture processes. Consequently, the development of reliable and accurate tools to predict mixture adsorption data is required to link materials’ adsorption properties to their process performance.

So far, in computational screening studies, the prediction of mixture adsorption isotherms is usually done by fitting the simulated isotherms for the pure components to one specific mixture adsorption model. Moreover, for process optimization purposes, mixture adsorption data is required at many different operating conditions. It is therefore important that one selects the right adsorption isotherm model such that the data can be described sufficiently accurately. Likewise, if experimental data is used to describe the thermodynamics of an adsorption process, much effort is spent in fitting that data to the right model. This cumbersome fitting process cannot be utilized in screening studies of hundreds or thousands of materials, hence the need for a robust workflow that gives us an optimal prediction of the mixture adsorption isotherms for a wide range of pressures, temperatures, and compositions. In this workflow, we aim to achieve a sufficiently accurate description of the thermodynamics of the adsorption process, with the minimal number of simulation data for the pure components. This is a crucial step because the accuracy of our predictions heavily relies on the quality of the pure component isotherms, especially at low coverage.^17^ This low coverage regime is of particular importance for capturing CO_2_ from dilute sources such as confined spaces or directly from the air, where the CO_2_ concentration falls significantly below 1%. Several studies have shown that ideal adsorbed solution theory (IAST) accurately predicts binary CO_2_ and N_2_ adsorption isotherms for zeolites and metal–organic frameworks using computational data.^14,18−20^ In contrast, Gharagheizi and Sholl^21^ concluded from a set of experimental mixture data that, for CO_2_/N_2_ mixtures, IAST predictions are the worst for all mixtures considered in their study. These contradicting arguments motivated us to carry out a careful study on the reliability of predicting mixture adsorption isotherms at conditions of interest for carbon capture applications.

In this paper, we present a workflow that is designed to work without manual intervention to efficiently predict, by using molecular simulations, the thermodynamic data that is needed to design a carbon capture process. We developed a procedure that does not rely on fitting the adsorption isotherms. From molecular simulations, we can obtain accurate data for both the pure component isotherms and the mixture isotherms. This allowed us to make a detailed comparison of the different methods to predict mixture isotherms. All approaches rely on an accurate description of the pure component isotherms and a model to predict the mixture isotherms.^22^ As we are interested in low CO_2_ concentrations, it is essential that these models correctly predict the low-pressure limit, i.e., give a correct description of the Henry regime. Among the equations that describe this limit correctly, the dual-site Langmuir (DSL) model^23^ is often used for the pure components and the extended DSL (EDSL) is used for the mixtures. An alternative approach, which avoids describing the pure component isotherms with a model, is to numerically integrate the pure component isotherms in the context of IAST.^24^ In this work, we compare these two methods. In addition, we show that the way these data are fitted for DSL can significantly impact the ranking of materials, in particular for capture processes with a low concentration of CO_2_ in the feed stream.

## Simulation Details

In this work, we focus on metal–organic frameworks (MOFs). MOFs are crystalline materials, and by changing the linker one can potentially synthesize over 1 trillion different materials. Much research has been focused on finding the optimal MOF for carbon capture.^25−28^

Ideally, one would like to have a set of experimental pure components and mixture isotherms to test the approach we developed here. However, the number of MOF structures for which accurate mixture adsorption isotherms have been experimentally measured over a large range of pressures, temperatures, and compositions is very limited, especially for a mixture of CO_2_ and N_2_.^29^ Therefore, we rely on molecular simulations to predict the adsorption isotherms of both the pure components and their mixture. Here, we use a set of 500 MOF structures that were chosen to represent a diverse set of metals, linkers, ligands, pore sizes, and topologies.^30^

In our molecular simulations, we assumed that the crystal structures are rigid. First, each crystal structure was cleaned (i.e., atomic overlaps were removed, solvent molecules were removed, and missing hydrogen atoms have been added) and subjected to density functional theory (DFT) geometry optimization. The DFT electronic density was also used to compute partial charges via the DDEC protocol.^31^

The TraPPE^32^ force field was used to describe the CO_2_–CO_2_, N_2_–N_2_, CH_4_–CH_4_, CO_2_–N_2_, and CO_2_–CH_4_ interactions. H_2_–H_2_ interactions were described by the Buch^33^ potential without Feynman–Hibbs rules (Buch-nonFH), for a simpler use with mixtures. For the CO_2_–H_2_ and guest–MOF interactions, the TraPPE-(Buch-nonFH) and the TraPPE-UFF^34^ force-field combinations were selected respectively, as these are common force fields adopted by the community. In the literature, one can find other force fields that give comparable descriptions of the pure component isotherms. As we focus in this work on how well IAST can predict mixture isotherms, we did not investigate in detail the accuracy of the force fields for the different systems.

We used Monte Carlo simulations in the canonical ensemble of one molecule to compute the heat of adsorption at infinite dilution, Δ*H*_H_ (kJ mol^–1^), and Widom test particle insertions to compute the Henry coefficient, *K*_H_ (mol kg^–1^ bar^–1^).^35^ Complete adsorption isotherms were computed using grand canonical Monte Carlo (GCMC) simulations.^35^

All the calculations were run using the Automated Interactive Infrastructure and Database for Computational Science, AiiDA.^36^ Pure component isotherms were obtained using the associated AiiDA workflow named IsothermAccurateWorkChain, which is part of the aiida-lsmo plugin.^37^ Being part of the same plugin, MulticompGcmcWorkChain was the workflow used to calculate the binary GCMC data.

In addition to all the isotherm calculations, the heat capacities of the different MOF structures were estimated using our newly developed machine learning method,^38^ which simply uses the crystal structure as an input. These heat capacities are used later on as inputs to the process model. More details on the structures, the force fields, and simulations can be found on the Materials Cloud^39^ and in the Supporting Information.

## Pure Component Isotherms

If one screens a very large number of structures, it is not feasible to manually decide on the pressures for which the loading is computed from a molecular simulation. One can, of course, use a brute-force method, in which we simulate such a large number of pressure points that we are guaranteed to have a correct sampling for all materials. However, with limited computational resources, it is important to develop a workflow that ensures an efficient sampling for all materials.

### The Computational Workflow

We carry out the computation of an isotherm in two steps. In the first step, we compute the Henry coefficient of all materials. Only for those materials that have a sufficiently high Henry coefficient for CO_2_, we will compute the complete isotherm. In the Henry regime, the isotherm follows from
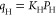
1where *q*_H_ is the loading (mol kg^–1^), *K*_H_ is the Henry coefficient, and *P*_H_ is the pressure for which we start to observe significant deviations from the Henry regime. As we have computed the Henry coefficient accurately for all materials, we only need to simulate the complete adsorption isotherm from the pressure (*P*_H_). To estimate *P*_H_, we used the following steps:

• As a first guess of the limit of the Henry regime, we assume to have reached *P*_H_ at a loading of one molecule per unit cell of the crystal. If Henry’s law holds, this pressure follows from
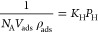
2where *V*_ads_ is the volume of the unit cell (m^3^), ρ_ads_ is the density of the unit cell (kg m^–3^), and *N*_A_ is Avogadro’s number.

• The true uptake, *q*_H_, is obtained by running a GCMC simulation at *P*_H_. If *P*_H_ belongs to the Henry regime, the error between the true uptake and Henry’s uptake should be smaller than a set precision value, ϵ, as
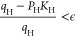
3

• If the error is higher than ϵ, we have overestimated the pressure for which the Henry regime holds, and *P*_H_ is decreased by a factor of 0.8. Also, we repeat the previous steps until the error converges to a value smaller than ϵ.

It is important to predict Henry’s coefficients with high accuracy. However, we would like to avoid running the protocol described by eq 3 indefinitely. Therefore, we allow for a 7.5% error margin compared to the ones obtained by running the Widom Insertion technique.

For pressures above *P*_H_, we use GCMC simulations to compute the isotherms. If we approach the saturation loading, these GCMC simulations become very time-consuming as the probability to insert an additional molecule becomes extremely low. In addition, under these conditions, the pressure is much higher than we typically reach in practical applications. Therefore, we stop our GCMC simulations at a pressure *P*_sat_, where we have theoretically reached 95% of the estimated maximum loading. We have tested higher loading settings, but, except for increasing the CPU requirements, a higher value did not significantly change the results.

To estimate *P*_sat_, we assume that we can describe the adsorption isotherm with a Langmuir isotherm:
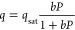
4where *b* is the adsorption equilibrium constant and can be related to the Henry coefficient. For *P* → 0, the Langmuir model describes the Henry regime, hence
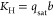
5

At 95% of the maximum loading, the Langmuir approximation gives
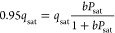
6Rearranging the equation to solve for *P*_sat_ gives
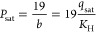
7where *K*_H_ has been computed previously. To compute *q*_sat_, we use the procedure described by Ongari et al.^37^ In this procedure, it is assumed that, at saturation, the density of adsorbed molecules, ρ_liquid_^guest^, is equal to the liquid density:
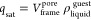
8where *V*_frame_^pore^ (m^3^ kg^–1^) is the probe-occupiable accessible pore volume^40^ of the crystal structure and can be computed from the crystal structure by Zeo^++^.^41^ For the liquid densities of the guest molecules, we used 25.02 and 28.84 (mol m^–3^) for CO_2_ and N_2_, respectively. By substituting eq 8 into eq 7, we can compute *P*_sat_ using
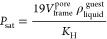
9

GCMC simulations are now performed only on the relevant part of the isotherm, i.e., from *P*_H_ to *P*_sat_. A further gain in computing efficiency can be obtained if we adjust the number of pressure points to the steepness of the isotherm; the steeper the isotherm the smaller the steps in pressure. To achieve this, the pressure points are selected based on the formula^37^
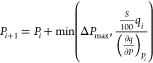
10The second term, containing the derivative, ensures that the change in uptake does not exceed *S*%. *S* can be selected based on how smooth the sampling must be. The higher the value of *S*, the lower the number of pressure points. The maximum pressure step (Δ*P*_max_) is determined so that in the worst-case scenario *N* points are generated as
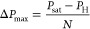
11

The derivative of uptake with respect to pressure is obtained numerically by applying the backward divided difference scheme:^42^
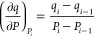
12The uptake is obtained by running GCMC calculations for each pressure point. The heat of adsorption is also an output of these calculations. Given that the uptake is an increasing function of pressure, the derivative must always be positive. In the case where this is not true, the last run at *P*_*i*_ is always repeated.

**Figure 1:**
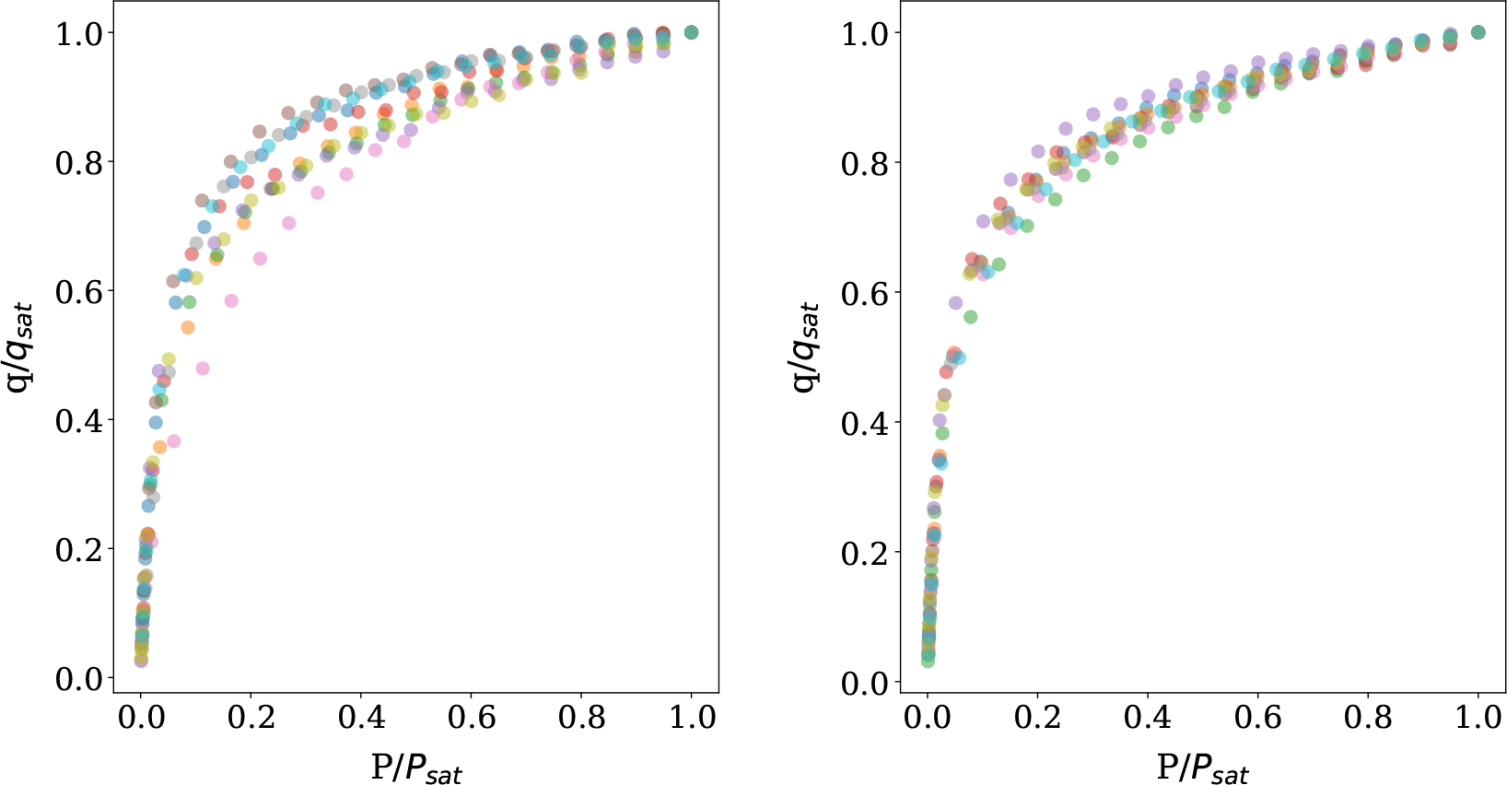
Pure component isotherms at 25 °C for CO_2_ (left) and N_2_ (right). The plots show the reduced uptake as a function of reduced pressure for the exemplar group of 10 structures.

Figure 1 shows the pure component isotherms of CO_2_ and N_2_ computed at 25 °C for an exemplar group of 10 structures. In the case of CO_2_, the workflow successfully predicted saturation, where the reduced uptake is shown to converge to 1, i.e., plateauing when the reduced pressure approaches 1. In the case of N_2_, higher pressures are required to reach saturation. However, *P*_sat_ values are beyond interest and it has no additional value to continue further. Finally, we use *N* = 25, hence 25 points per isotherm to ensure a smooth sampling of the pressure points. The workflow was later tested on the original set of 500 structures and was found to be reliable irrespective of the structure or the guest molecule, i.e., CO_2_ and N_2_, under investigation.

### Clausius–Clapeyron Relation

In pure component adsorption, two phases coexist at equilibrium in the system: the (ideal) gas phase and the adsorbed phase. In the same way, the Clausius–Clapeyron relation is used to relate temperature and pressure along phase boundaries in a phase diagram; it can also relate temperature and pressure for pure component adsorption systems.^43^ For adsorption, the Clausius–Clapeyron relation states that for two different isotherms, *T*_ref_ and *T*_new_, the same loading is obtained at two different pressures, *P*_*i*_^ref^ and *P*_*i*_^new^, respectively:
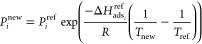
13where Δ*H*_ads_*i*__^ref^ is the heat of adsorption at *T*_ref_ and *P*_*i*_^ref^. Hence, the Clausius–Clapeyron relation can be used to predict pure component isotherms at different temperatures, given a reference isotherm. At the reference temperature *T*_ref_, our GCMC simulations at a pressure point *P*_*i*_^ref^ result in the corresponding uptake, *q*_*i*_^ref^, and heat of adsorption, Δ*H*_*i*_^ref^. The Clausius–Clapeyron relation allows us to compute at which pressure *P*_*i*_^new^ the same amount is adsorbed (*q*_*i*_^ref^ = *q*_*i*_^new^) but at a different isotherm, *T*_new_. Refer to Figure S1 for a visual description.

In eq 13 we have assumed that the heat of adsorption is a constant with respect to temperature, which is a valid assumption for a small temperature difference with respect to the reference temperature, i.e., 25 °C. In the Supporting Information, we choose a subset of 50 structures, in which we have made sure to have a variety of metals, linkers, and pore sizes. We use the Clausius–Clapeyron relation to extrapolate to four different temperatures: 50, 75, 100, and 125 °C. Then we use IAST to predict binary adsorption data of CO_2_ and N_2_ at those temperatures, for a wide range of CO_2_ compositions (0.04–95%). Finally, the predictions are compared to the binary data computed using the pure component isotherms generated by GCMC calculations at the respective temperatures. We are able to show that, if we compute the reference CO_2_ and N_2_ isotherms at 25 °C, we can obtain, for the majority of the predictions, binary CO_2_ and N_2_ data within an accuracy of 1–3%. The higher the temperature difference with respect to the reference temperature, i.e., 25 °C, the higher the errors, which corroborates the assumption that a constant heat of adsorption is only valid for small temperature differences. In the extreme case of 125 °C, very few predictions are calculated with errors up to 25%. One way to overcome this issue is to use another reference isotherm at a relatively higher temperature, 75 °C for example, in order to minimize the temperature difference when extrapolating at high temperatures.

## Binary Adsorption Isotherms

The number of published experimental binary CO_2_ and N_2_ adsorption isotherms in MOFs is very limited.^29^ For a pure component isotherm, one can measure the weight increase as a function of pressure, while for a mixture, one also has to measure the relative concentration in the pores. Hence, for most practical applications we have to rely on the predictions of binary adsorption isotherms using the pure component isotherms as input.

Unlike experiments, in a GCMC simulation, the computation of a binary adsorption isotherm is similar to that of a pure component isotherm. But, for most practical applications one has to determine the isotherms for (many) different compositions, making this a computationally expensive step.

In the literature, different routes are used to predict binary adsorption isotherms. In this section, we give a short summary of the different methods. For a more extensive review we refer to the literature.^44^

### Ideal Adsorbed Solution Theory

Ideal adsorbed solution theory (IAST) is a thermodynamic theory that is applied in this study for predicting mixture adsorption data from calculated pure component isotherm data at a given temperature.^45^ The method relies heavily on the thermodynamics of physical adsorption, and its accuracy depends on four main assumptions:

1. Adsorbates have equal access to the entire surface area of the adsorbent.

2. During the adsorption process, changes in the thermodynamic properties of the framework are negligible compared to the property changes of the adsorbable species.

3. The adsorbed phase is described by the Gibbs energy of adsorption. One simply substitutes the spreading pressure (π) for pressure (*P*) and area (*A*) for volume (*V*):
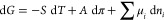
14

4. The vapor phase is considered an ideal gas and the adsorbed phase an ideal solution.

With these assumptions, the adsorbed gases can be described as an ideal gas mixture in equilibrium with an ideal adsorbed phase on the adsorbent surface. For a binary mixture of, say, CO_2_ and N_2_, and for a given temperature, a given gas composition, and the total gas pressure, the number of moles adsorbed for a binary mixture is obtained by solving simultaneously the set of equations governing IAST.^45^ One of these IAST equations follows from the condition that, at equilibrium, the spreading pressures of CO_2_ and N_2_ are equal:
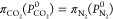
15a
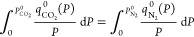
15b
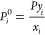
15c

Here *q*_*i*_^0^ is the pure component isotherm, *P* is the total pressure, *y*_*i*_ and *x*_*i*_ are the gas phase and adsorbed phase compositions respectively, and *i* can be either CO_2_ or N_2_. Finding a solution for eq 15b involves solving integrals. Mathematically, integrals can be solved either analytically, if the pure component isotherm has a functional form (*q*_*i*_^0^ = *f*(*P*)), or numerically if it does not. Simon et al.^24^ developed a Python package, the ideal adsorbed solution theory Python (pyIAST) package, that supports both options. Pure component isotherms can either be fitted to thermodynamically consistent models, such as Langmuir, dual-site Langmuir, quadratic, BET, Henry’s law, and approximated Temkin, or be linearly interpolated in order to calculate the integral using numerical quadrature. In this work, we use the numerical option of pyIAST. The numerical option has the practical advantage that the outcome does not depend on the functional form that is used to describe the experimental or computational adsorption isotherm. This agrees with the work of Chen and Sholl,^46^ where they introduce a new Monte Carlo technique and combine it with IAST to avoid curve fitting.

### Extended Dual-Site Langmuir Model

The DSL isotherm model was introduced in an attempt to describe nonideal adsorbate–adsorbent interactions. In this model, it is assumed that the deviations from ideality are due to surface heterogeneity.^23^ The model consists of two sites, 1 and 2, that are independent of each other, where each site follows the Langmuir equation. Hence, the dual-site adsorption of a pure component is simply the sum of adsorption on each site:
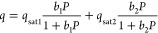
16It is important to note that this model meets the thermodynamic consistency requirements for pure gas adsorption;^47^ i.e., at infinite dilution, the limiting slope of the isotherm is positive and finite, the spreading pressure is a continuous and well-behaved function of pressure, and the isosteric heat of adsorption at constant uptake is finite.

The DSL model can be extended to predict a binary mixture isotherm. If we consider a mixture of gases A and B, the corresponding extended DSL model for a binary mixture is described as^23^
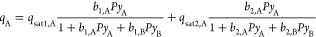
17a
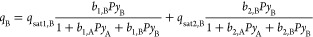
17b

It is important to note that, for the extended model to be thermodynamically consistent, the saturation capacities for both components must be equal; therefore^23^
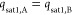
18a
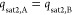
18b

To generate the binary adsorption data, the parameters of the extended Langmuir model are obtained from the fitting of the pure component isotherms to the DSL model. In some cases, the number of data points within the pure component isotherm is insufficient for fitting purposes. To generate more data points, we applied linear interpolation to the original pure component isotherms.

The parameters of the DSL model are sensitive to the fitting procedure used. Consequently, process modeling results will also be affected.^48^ We followed the fitting procedure discussed by Farmahini et al.^48^ to ensure thermodynamic consistency, which involved minimizing the least-squares error between the simulated isotherms and the ones predicted by the model.

## Results

For the pure component isotherms, we observed that the fitting procedure to obtain the DSL parameters is sensitive to initial guesses, i.e., different initial guesses lead to different model parameters. For example, Figure S4 shows the results of exemplar fits. In the region where we have abundant data, the different fits give equally good descriptions. This is not surprising as many combinations of parameters give comparable results. However, for applications where the CO_2_ concentration is very dilute, it is important that the model accurately covers the Henry regime. Figure S5 also shows that the different initial guesses give very different predictions for the low-pressure regime. In some cases, it was impossible to obtain a meaningful fit that covers both low- and high-pressure regions simultaneously. Therefore, the binary predictions at low or high pressures, depending on where we put the weight on the fit, will be compromised (see Figure S5). In addition, if we fit the data we are not guaranteed to obtain values of the DSL parameters that are physically reasonable. For example, in eq 16*q*_sat1_ and *q*_sat2_ are directly related to the saturation uptakes, but the values obtained from the fits do not match the ones from the pure component isotherms. In Figure S6 we show how different these values are. Higher differences are observed in structures for which the saturation takes place way beyond 10 bar. Therefore, the fitting is sensitive to the range of data being used.

In a few cases, the fitting procedure completely fails or the obtained parameters are physically inexplicable. The predicted saturation uptake is on the order of 1000 mol kg^–1^, or the heat of adsorption is a positive value. In those cases, the pure component isotherms were accurately described by the DSL model mathematically; however, thermodynamically, the parameters are not valid and the fits cannot be trusted (see Figure S7). Generally, not every isotherm can be fitted to a DSL model, and assuming that, in a set of pure component isotherms for different materials, all isotherms follow the DSL model, or any other model, can be erroneous.

In all these cases, we also compared the predictions of the mixture isotherms with simulation results. The results are shown in Figure S8. The direct integration of the pure component isotherms in the IAST framework avoids all these fitting problems and gives reasonable results for the mixture isotherms.

**Figure 2:**
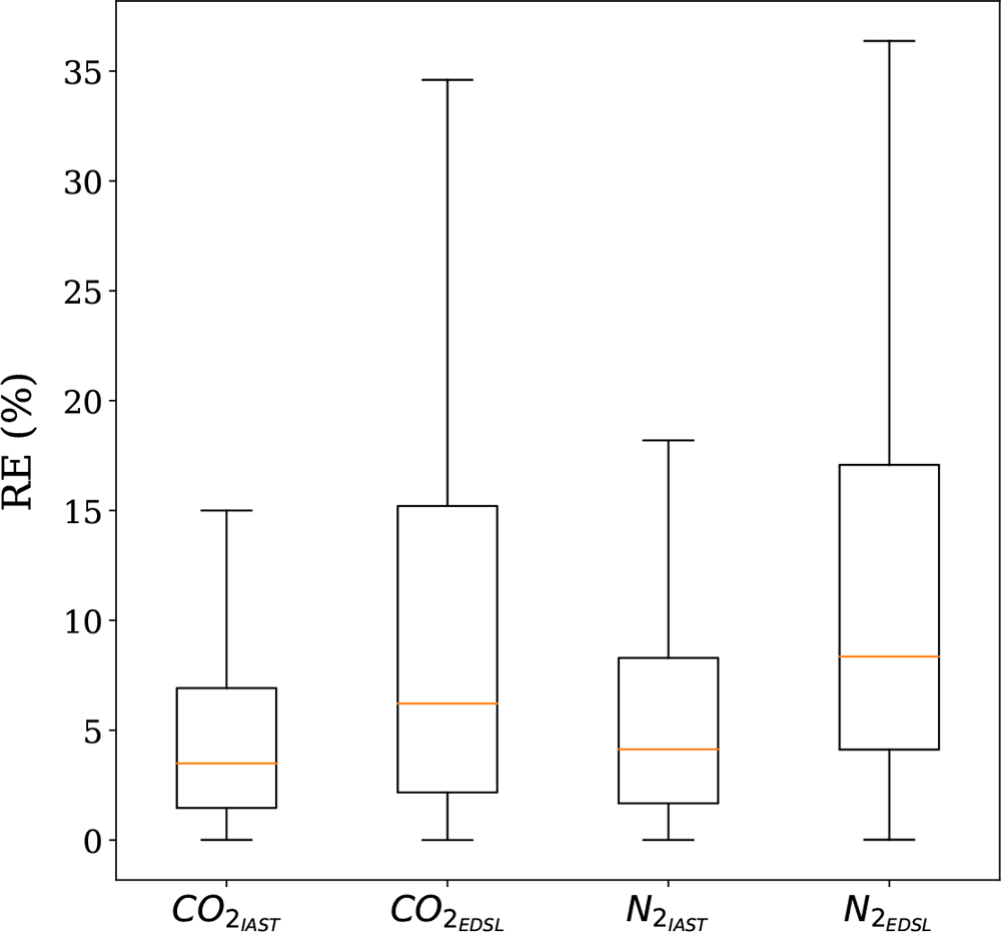
Relative error distributions for all conditions and 50 structures. The relative errors were obtained using RE_*i*_ = 100|*q*_*i*,IAST/DSL_ – *q*_*i*,GCMC_|/*q*_*i*,GCMC_. In these box plots, the middle line in the box divides the data set in two: 50% of the data has an error smaller than the one corresponding to the line and 50% has an error larger. The region between the lowest whisker and the bottom of the box contains 25% of the data with the lowest error, while the top of the box and the upper whisker contain the 25% of the data with the highest error. These box plots are shown without outliers, i.e., data points that are located outside the whiskers of the box plot.

To quantify the differences in accuracy of the predictions between IAST and extended DSL, we compared the predicted and simulated (GCMC) CO_2_ and N_2_ mixture isotherms for a range of temperatures (25–125 °C) and CO_2_ compositions (0.0004–0.95) and a fixed pressure (1.01325 bar) for the subset of 50 structures. All data points were put together, and we used box plots to visualize the error distribution as shown in Figure 2.

When we compared the relative errors, IAST predictions were closer to the binary GCMC calculations than the predictions given by the extended DSL model. This was true for all the different conditions, structures, and guest molecules. One can see that the median relative error for both molecules, using IAST, is less than 5% compared to 10% when extended DSL is used. In each case, we had 1250 data points and the outliers counted for almost 10%. These outliers represent the data lying outside the whiskers of the box plot. However, some of them are due to inaccurate binary GCMC calculations, which are usually at very low pressures. From these results, one can conclude that IAST provides good predictions of binary adsorption data for the set of diverse structures over the temperature and CO_2_ partial pressure ranges of interest. These results are of no surprise. It was previously established, in multiple studies,^14,18,19^ that IAST accurately predicts binary CO_2_ and N_2_ adsorption isotherms for zeolites and MOFs using computational data.

**Figure 3:**
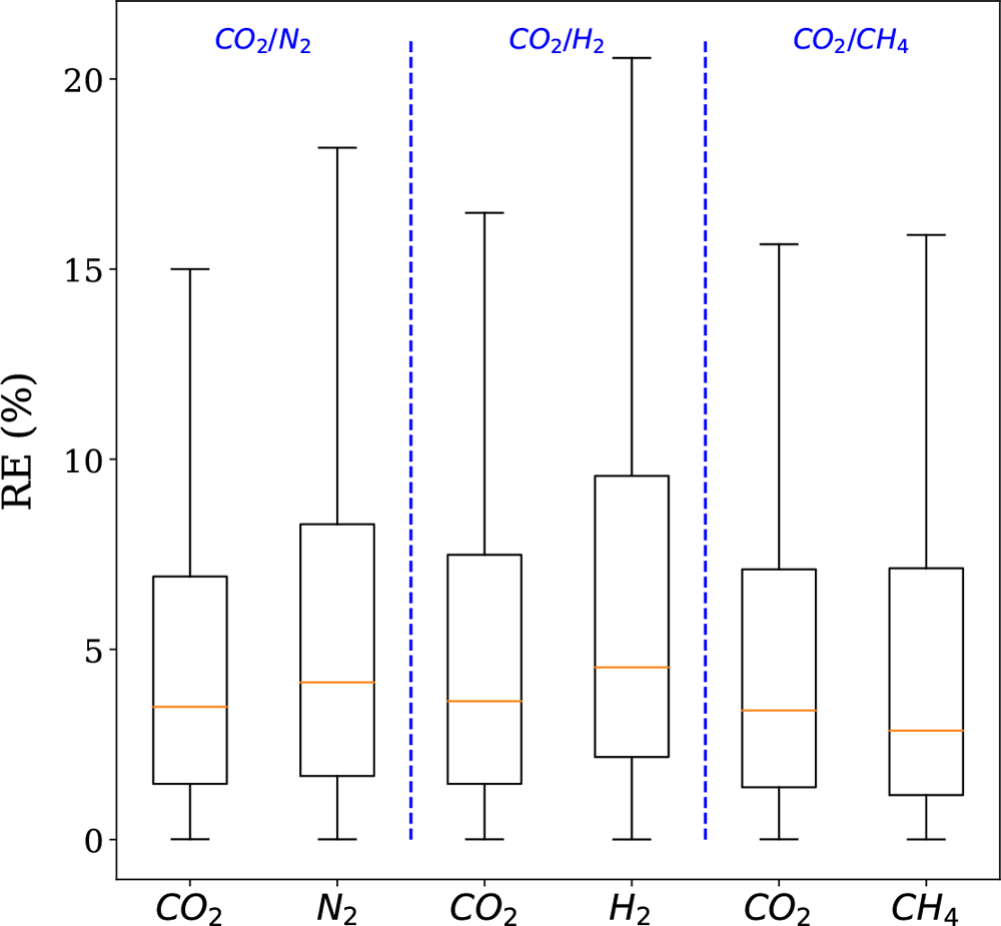
Relative error distributions for all conditions and three different mixtures, CO_2_/N_2_, CO_2_/H_2_, and CO_2_/CH_4_. The relative errors were obtained using RE_*i*_ = 100|*q*_*i*,IAST_ – *q*_*i*,GCMC_|/*q*_*i*,GCMC_. See also the caption to Figure 2.

We further investigated whether similar conclusions hold for other CO_2_ mixtures, i.e., CO_2_/H_2_ and CO_2_/CH_4_. We used the same subset of 50 structures. We used the same procedure as for CO_2_/N_2_ to compare the IAST predictions to the simulated (GCMC). Figure 3 shows that IAST predicts CO_2_ binary uptake with almost the same accuracy for the three different mixtures. Considering the other component in each mixture, IAST predictions are slightly better for CH_4_, and we observe a slightly larger error bar for H_2_. Overall, 75% of IAST predictions lie within 10% error.

### Importance of Isotherm Sampling

Gharagheizi and Sholl^21^ concluded in their study of the reliability of predictions of mixture isotherms that, for a CO_2_/N_2_ mixture, IAST gave the worst results. This conclusion appears to be in sharp contrast to our conclusion that IAST performs the best. In this section, we show that the work of Gharagheizi and Sholl points at an important limitation of the IAST theory, which becomes apparent if one uses the pyIAST^24^ package.

The pyIAST package was developed with the idea that mixture isotherms are computed directly from the experimental (or simulated) pure component isotherms, without the need for any fitting. In our workflow to compute the pure component isotherms, we have ensured that we obtain adsorption isotherms up to 95% of the saturation loading, where we estimate the saturation loading from the pore volume using the liquid density of the adsorbent. In most cases, the liquid density results in an overestimation of the saturation uptake. Hence, our calculations require more CPU time than strictly needed. The consequence of underestimating the saturation loading is studied in detail later in this section, and on the basis of the results, we concluded that computing the isotherms up to 95% of the saturation provides a sufficiently large safety margin.

**Figure 4:**
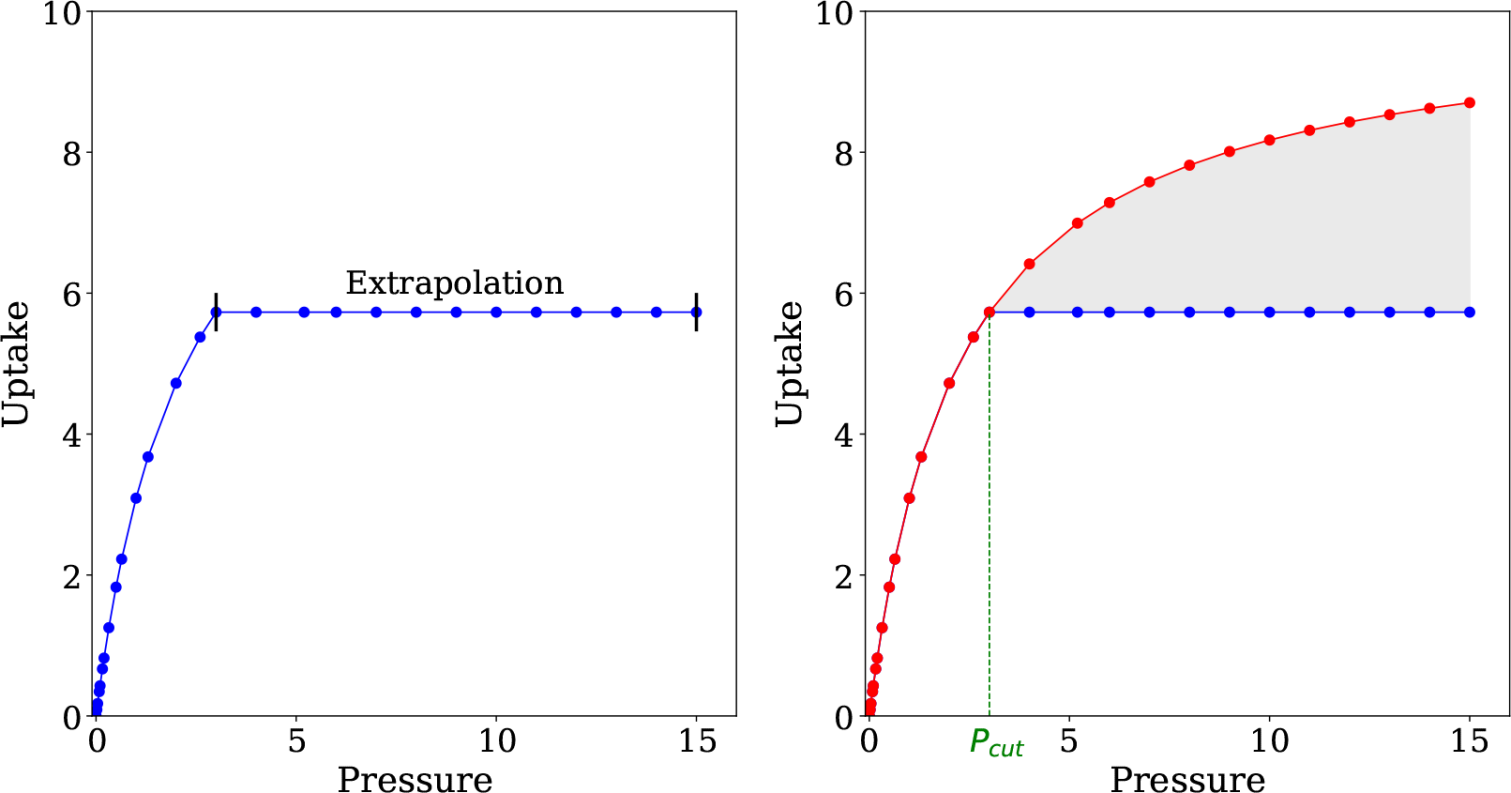
Extrapolating beyond the pressure limit for an incomplete isotherm using pyIAST (left). The difference between a complete and an extrapolated incomplete isotherm (right).

Experimentally, in particular for N_2_, one often has isotherms that have been measured up to a maximum pressure that is far from saturation. In the case of such an incomplete isotherm, one has to be careful with pyIAST. The advantage of the pyIAST package is that we obtain the optimal IAST prediction given the experimental (or computational) data that is provided, and we do not have to rely on a fitting step. However, this also implies that pyIAST is unable to extrapolate to pressures higher than are provided. pyIAST extrapolates beyond the last pressure point by assuming that saturation was reached and the loading is a constant, equal to the largest loading available in the pure component data. The pyIAST package gives a warning that it had to use extrapolated data, but it cannot give an indication of the error this might give. This is best illustrated in the left-hand side of Figure 4.

Given that IAST calculations are performed using numerical quadrature to compute the spreading pressure, it is important to understand how “extrapolating” beyond the last pressure point using pyIAST affects the mixture predictions. On the right-hand side of Figure 4, we consider a complete pure component isotherm (red line), which is then truncated at *P*_cut_. pyIAST is used to extrapolate the resulting incomplete isotherm (blue line) as described previously. The shaded area corresponds to the integration difference between the complete and incomplete isotherms and can be described by
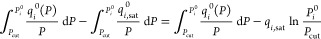
19where *P*_*i*_^0^ is defined in eq 15c and *i* can be either CO_2_ or N_2_. By definition, eq 19 is only valid for *P*_cut_ < *P*_*i*_^0^.

To obtain some insight into the importance of this truncation, we choose pure component isotherms from one of the exemplar structures shown in Figure 1. We mimic the case in which we can only have data up to a maximum pressure, which is a fraction of the saturation pressure, by truncating the corresponding CO_2_ and N_2_ pure component isotherms at different pressures. Those isotherms are then used along with pyIAST to predict binary data at 25 °C, 1.01325 bar, and different gas compositions (10 and 90% CO_2_). The binary data predicted using the extrapolated incomplete isotherms is compared to the binary data predicted using the complete pure component isotherms, by computing the relative errors for CO_2_ and N_2_.

The saturation uptake of the structure in question is 4.7 mmol g^–1^, and it is reached at 2 and 246 bar for CO_2_ and N_2_, respectively. Using the complete pure component isotherms, we predict *P*_CO_2__^0^ and *P*_N_2__^0^ to be equal to 0.1 and 14 bar respectively for a mixture of 10% CO_2_. For a mixture of 90% CO_2_, *P*_CO_2__^0^ and *P*_N_2__^0^ are equal to 0.9 and 126 bar, respectively. We observe that increasing the composition of CO_2_ will increase the *P*_*i*_^0^ values by a similar factor, in this case by a factor of approximately 9.

**Figure 5:**
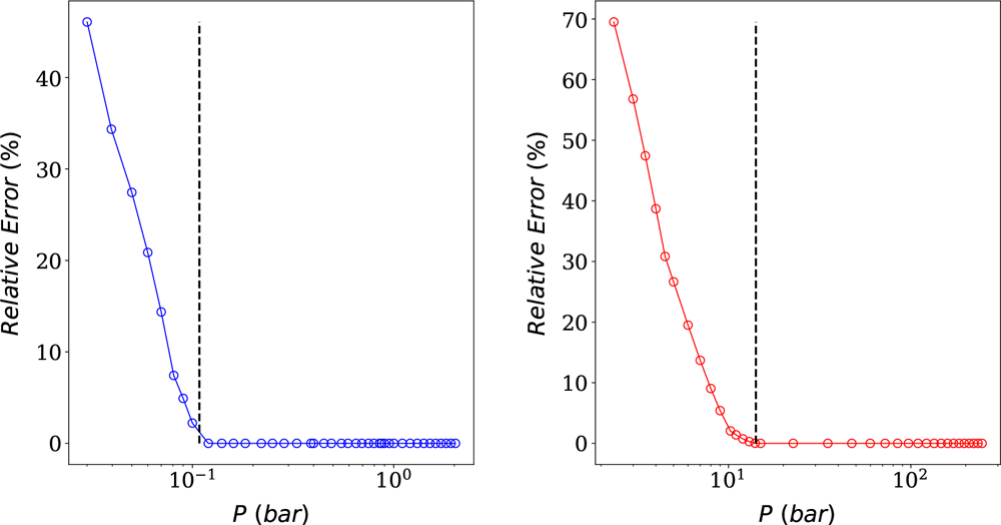
Plots of relative errors in IAST predictions as a function of the truncated pressure for CO_2_ (left) and N_2_ (right) for a mixture of 10% CO_2_. The dashed lines correspond to *P*_*i*_^0^ values at the given mixture composition.

**Figure 6:**
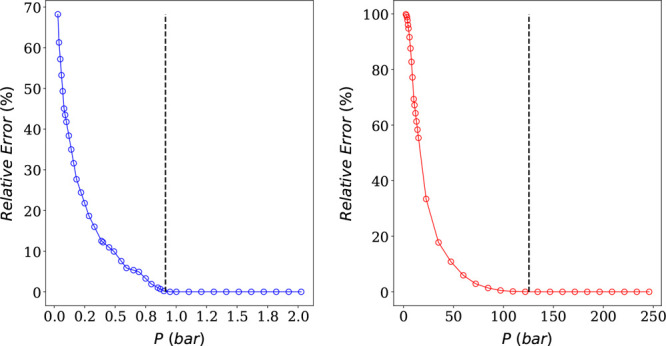
Plots of relative errors in IAST predictions as a function of the truncated pressure for CO_2_ (left) and N_2_ (right) for a mixture of 90% CO_2_. The dashed lines correspond to *P*_*i*_^0^ values at the given mixture composition.

Figures 5 and 6 show the effect of truncating the CO_2_ isotherm on CO_2_ IAST predictions keeping the N_2_ pure component isotherm intact (left), as well as the effect of truncating the N_2_ isotherm on N_2_ IAST predictions keeping the CO_2_ pure component isotherm intact (right), for mixtures of 10 and 90% CO_2_, respectively. It is evident from both figures that, if the pure component isotherm of component *i* is truncated at a pressure higher than *P*_*i*_^0^, the IAST predictions are exact and the relative error is always null. This follows from the definition of eq 19. Nevertheless, if the truncated pressure lies to the left of *P*_*i*_^0^, the pure component isotherm loses “important information” and pyIAST’s extrapolation leads to incorrect mixture predictions. Also, the further we truncate to the left, the higher the relative error. In fact, there is an exponential correlation between the relative error and the truncated pressure.

The value of *P*^0^ dictates how accurate the sampling of a pure component isotherm should be. However, this value cannot be predicted a priori. Our computational workflow offers a suitable solution and can be safely used in conjunction with pyIAST, as it generates CO_2_ and N_2_ isotherms up to the saturation point. Interestingly, N_2_ saturation is reached at relatively higher pressures compared to CO_2_. From an experimental perspective, complete N_2_ isotherms will be difficult to obtain due to the equipment limitations.

We can now understand why Gharagheizi and Sholl^21^ obtained the worst results with IAST, as they implicitly assumed by using pyIAST that the experimental N_2_ isotherms were obtained at sufficiently high pressures. However, most experimental N_2_ isotherms do not reach this pressure. If this is the case assuming a constant loading beyond the maximum pressure that is measured leads to large errors.

IAST predicts the mixture isotherms surprisingly accurately, but it can only do this at all compositions if it “knows” the pure component isotherms up to sufficiently high pressures. Fortunately, it is possible to extrapolate to these higher pressures if we know the pore volume of the material (e.g., from BET calculations) and then use eq 8 to approximate the saturation loading. With this approximation of the saturation loading and the low pressure experimental data, one can make a sensible extrapolation of the experimental data. If these data are then added to the experimental data, pyIAST will most likely give a much better prediction. Unfortunately, N_2_ isotherms are often overlooked and people tend to put more emphasis on CO_2_ isotherms.

## Process Modeling

**Figure 7:**
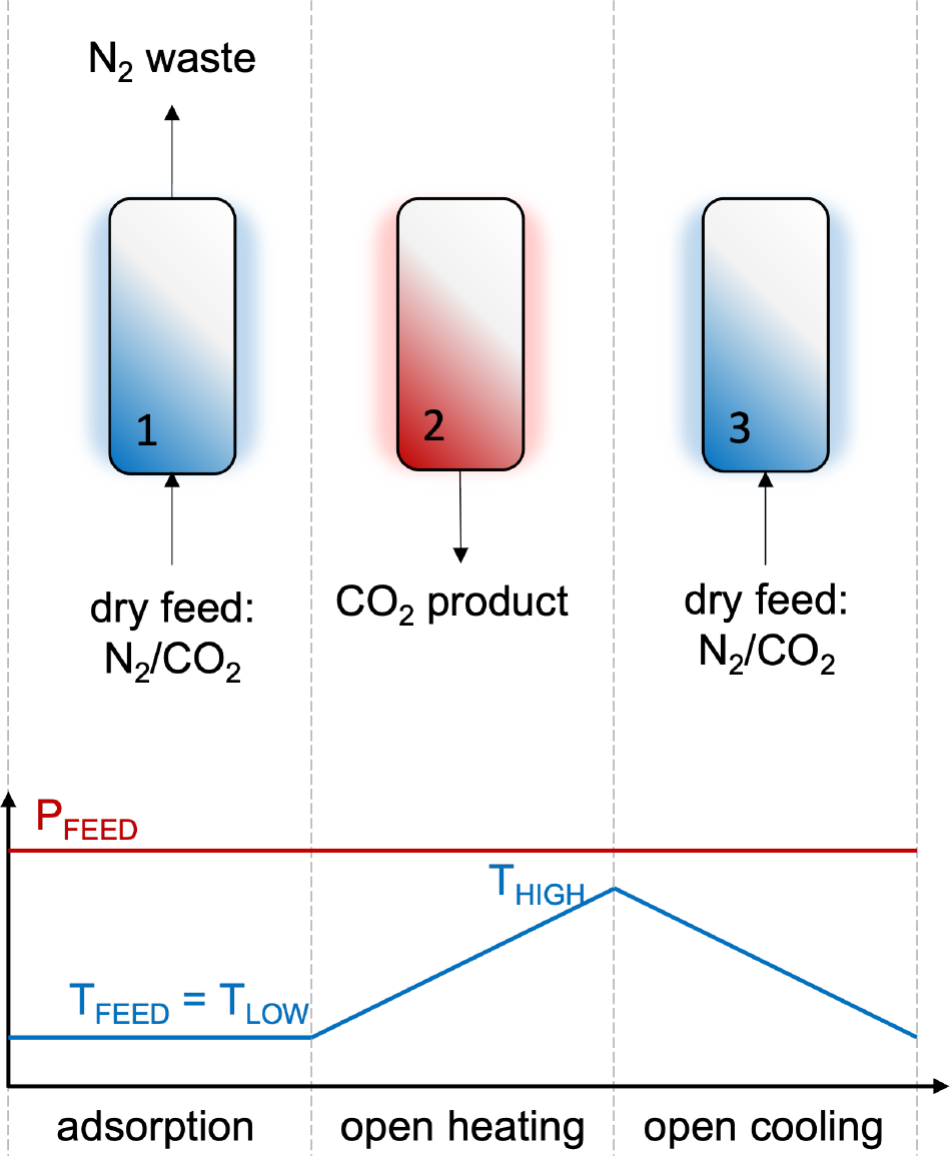
Standard three-step cycle TSA process: (i) adsorption, (ii) open heating, and (iii) open cooling.

In the previous section, we have shown that IAST gives a better prediction of the mixture adsorption isotherms compared to the extended DSL. In an industrial process simulation, the use of IAST requires much longer CPU times because of the integration, and because of this, preference is given to extended DSL or related equations which are numerically less demanding. These CPU times get even longer when we consider optimizing the process, given the multiple iterations involved. However, very little is known about how these differences in the thermodynamic description would impact the process design. For example, would a ranking of the performance of materials significantly change, or is the impact of these differences relatively minor? To answer this question, we consider an elementary temperature swing adsorption (TSA) process reproduced from the work of Ajenifuja et al.^49^ to capture CO_2_ from three different sources: (1) a coal-fired power plant (CF-PP), (2) a natural-gas-fired power plant (NG-PP), and (3) confined spaces (CS). This process is shown schematically in Figure 7.

**Figure 8:**
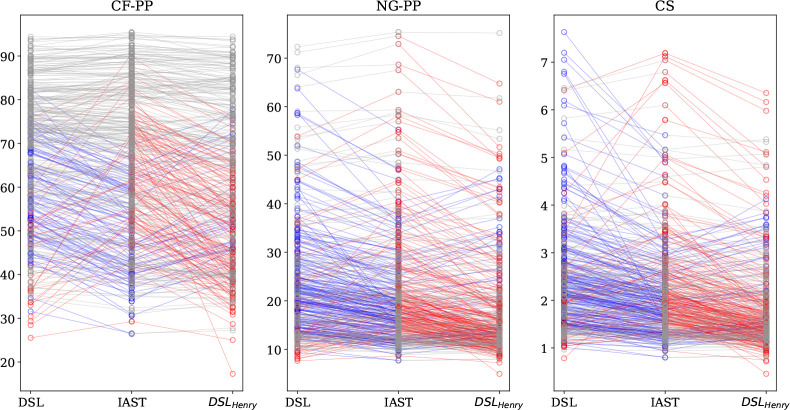
Purity (%) of output streams for a coal-fired power plant (CF-PP), a natural-gas-fired power plant (NG-PP), and confined spaces (CS). Each circle corresponds to a material where the middle gives the purity as predicted by IAST, and the left and right are from DSL, where in the right side more emphasis is put on fitting the Henry coefficient correctly. Points that correspond to the same material are connected by lines, where a gray line is used if the change between IAST and DSL or DSL_Henry_ is less than 10%, a blue line if the DSL gives 10% or higher purity, and red if it is 10% or lower. For visual clarity, a couple of data points were removed.

In evaluating the process performance, we considered two distinct fitting schemes: DSL and DSL_Henry_. In the first, all data points were given the same weight. In the second, we put more weight on the low-pressure region, such that we forced the DSL equation to describe the Henry coefficient correctly. In Figure 8 we show the materials ranking for a coal-fired power plant (CF-PP), a natural-gas-fired power plant (NG-PP), and confined spaces (CS). In Figure 8, we compare the purity (%) of CO_2_-rich product streams and we use the IAST results as the reference. For a fair comparison between IAST and extended DSL, 100 out of the initial 500 structures, for which the fitting procedure failed or the obtained parameters were physically inexplicable, were labeled as “problematic” and were discarded.

As can be expected, the range of purity that can be obtained in the process depends on the concentration of CO_2_ in the inlet gas and, in this case and for the evaluated TSA process, purity is lowest for the confined spaces. This is best illustrated in Figure 8. If we focus on the ranking of the materials in Figure 8, we see for the coal case that the top-performing materials all give similar purities, independently of the method that is used. However, if we go to natural gas or confined spaces, the order of the materials does depend on the method that is used, which corroborates the conclusion of the previous section that at low concentrations the differences between DSL and IAST can be significant. Interestingly, if we give more weight to the low-pressure regime, we tend to underestimate the purity (more red lines), and without this weight, we overestimate the purity (more blue). However, this trend is not true for all structures. Similar conclusions hold for other key performance indicators, such as the working capacity and specific heat requirements (see section 3.1 in the Supporting Information).

**Figure 9:**
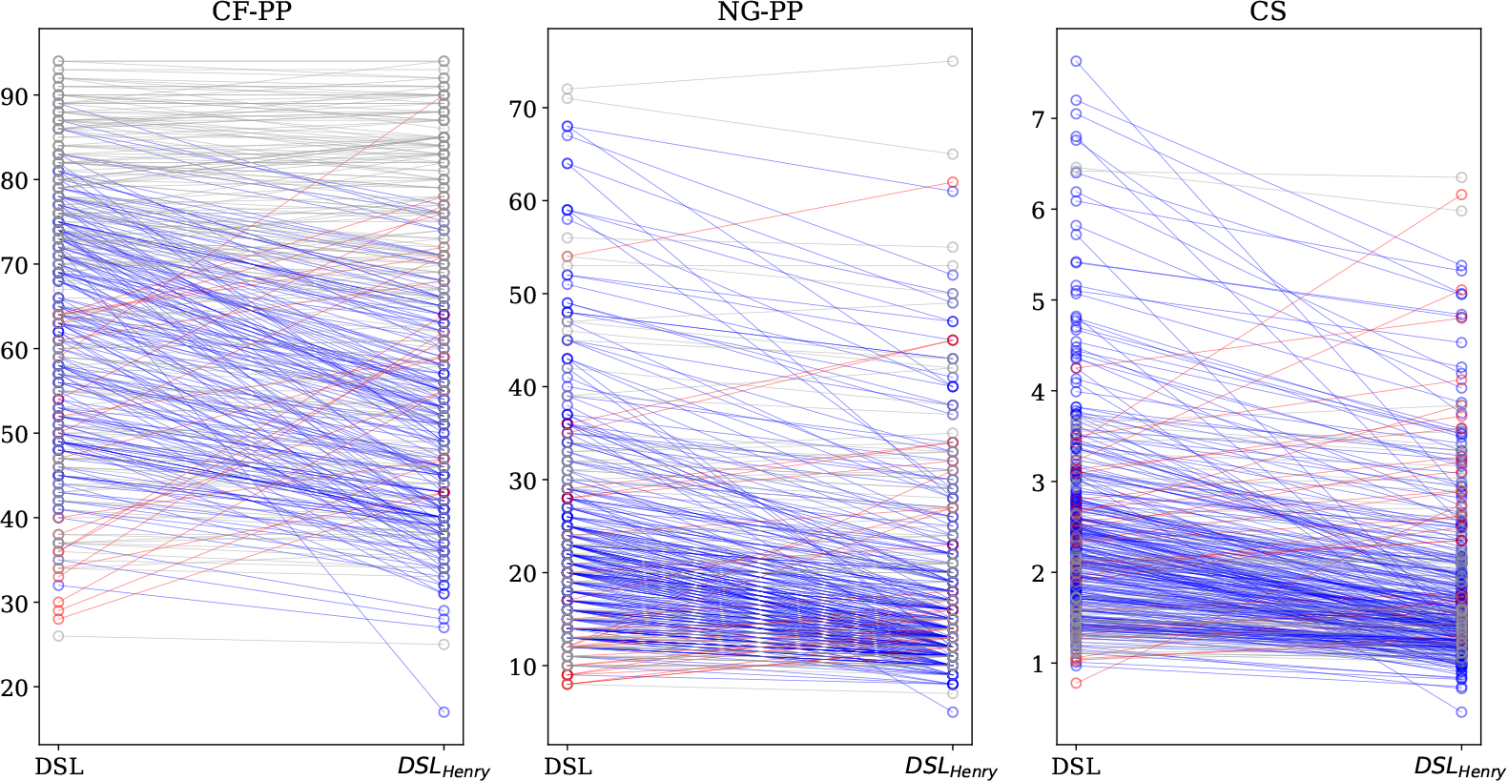
Purity (%) of output streams for a coal-fired power plant (CF-PP), a natural-gas-fired power plant (NG-PP), and confined spaces (CS). In this figure, we compare DSL with DSL_Henry_; see also the caption to Figure 8.

One important advantage of IAST is that we do not need to fit the data, while DSL does depend on how the data are fitted. In Figure 9 we illustrate the effect of the fitting procedure on the ranking. Figure 9 shows that DSL tends to give a higher purity than DSL_Henry_, but this does not hold for all structures.

**Figure 10:**
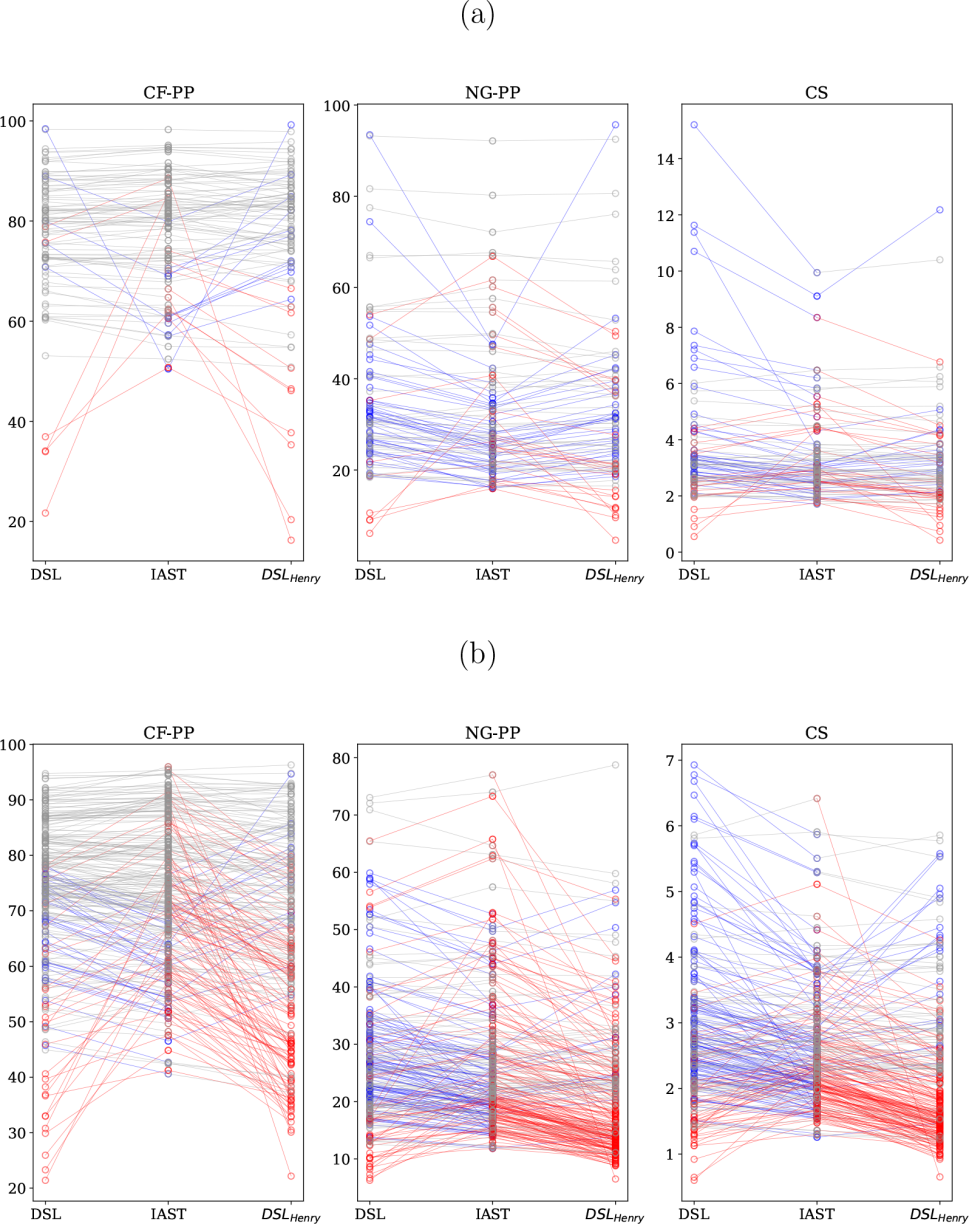
Purity (%) of output streams for a coal-fired power plant (CF-PP), a natural-gas-fired power plant (NG-PP), and confined spaces (CS) using isotherm data at pressures above 5 (a) and 25 mbar (b). The middle gives the purity as predicted by IAST, and the left and right are from DSL, where in the right side more emphasis is put on fitting the Henry coefficient correctly. See also the caption to Figure 8.

Another important aspect that is worth highlighting is that, in our simulations, we can obtain accurate data at very low pressure. Experimentally, however, the low-pressure regime is limited by the specifications of the available equipment in the laboratory. We use our data to estimate the impact of these experimental limitations in the low-pressure regime on the performance. For this, we consider two different scenarios. In the first, we assume that accurate data can be obtained above 5 mbar, and in a second scenario, we assume more routine experiments for which the minimum threshold was set at 25 mbar. In each scenario, we discarded the structures for which the CO_2_ and N_2_ reference isotherms did not have data points below the corresponding threshold, the fitting procedure failed, or the obtained parameters were physically inexplicable. We were left with 95 and 250 structures in each scenario, respectively. For those materials, it is important to realize the consequence of removing data points in the Henry regime. Figure 10 shows that the impact on the purity is higher when isotherm data only above 25 mbar was used. Some materials adsorb CO_2_ very strongly, and from those materials 25 mbar is already outside the Henry regime. We can also observe that the lower the CO_2_ concentration, the more the process performance is affected.

We can quantify how the ranking is changed by computing the Spearman coefficient, which is a measure of how much the ranking of the material is impacted. Table 1 shows the Spearman coefficients for the different cases. A Spearman coefficient of 1 implies that the ranking has not changed, while 0 indicates that the order has been completely lost.

**Table 1:** Spearman Coefficients Comparing the Ranking of Materials between DSL Fitting and IAST for a Coal-Fired Power Plant (CF-PP), a Natural-Gas-Fired Power Plant (NG-PP), and Confined Spaces (CS)^a^

	DSL	DSL_Henry_	DSL_5_	DSL_5Henry_	DSL_25_	DSL_25Henry_
CF-PP	0.91	0.85	0.84	0.79	0.89	0.74
NG-PP	0.86	0.81	0.86	0.85	0.81	0.86
CS	0.86	0.81	0.78	0.83	0.77	0.86

a The subscripts “5” and “25” indicate that the isotherm data below 5 and 25 mbar, respectively, have been removed from the data set to mimic the experimental limitations. The subscript “Henry” indicates that the fitting is done to ensure that the fits capture the Henry coefficient.

For a coal-fired power plant, we observe that the ranking of the materials is little affected by the method we use (Spearman coefficient larger than 0.85). Only if we lack isotherm data below 5 and 25 mbar and impose the fitting of the Henry coefficient, errors in our ranking become apparent (Spearman coefficients of 0.79 and 0.74, respectively). This is for cases for which we impose an incorrect Henry coefficient as the data for 5 and 25 mbar are already outside the Henry regime. For the natural gas case and confined spaces, the concentration of CO_2_ is so low that purity values are very sensitive to the method we use. However, the ranking of the material does not show significant deviations (Spearman coefficient larger than 0.8). Only in the case of confined spaces, if we lack isotherm data below 5 and 25 mbar, errors in our ranking become apparent (Spearman coefficients of 0.78 and 0.77, respectively).

The differences between DSL and IAST can cause some of the top-performing materials, as predicted by IAST, to not appear at the top for DSL. Table 2 summarizes the number of structures that appear in the top 40 performing materials as predicted by IAST for the different case studies using the DSL fitting. It is evident that one can miss from 10% in the best-case scenario to 33% of the top-performing structures when using extended DSL compared to IAST.

**Table 2:** Number of Structures That Appear in the Top 40 Performing Materials as Predicted by IAST for a Coal-Fired Power Plant (CF-PP), a Natural-Gas-Fired Power Plant (NG-PP), and Confined Spaces (CS) Using DSL Fitting^a^

	DSL	DSL_Henry_	DSL_5_	DSL_5Henry_	DSL_25_	DSL_25Henry_
CF-PP	35	34	35	31	32	29
NG-PP	29	27	34	35	32	30
CS	27	28	32	36	33	30

a The subscripts “5” and “25” indicate that the isotherm data below 5 and 25 mbar, respectively, have been removed from the data set to mimic the experimental limitations. The subscript “Henry” indicates that the fitting is done to ensure that the fits capture the Henry coefficient.

**Figure 11:**
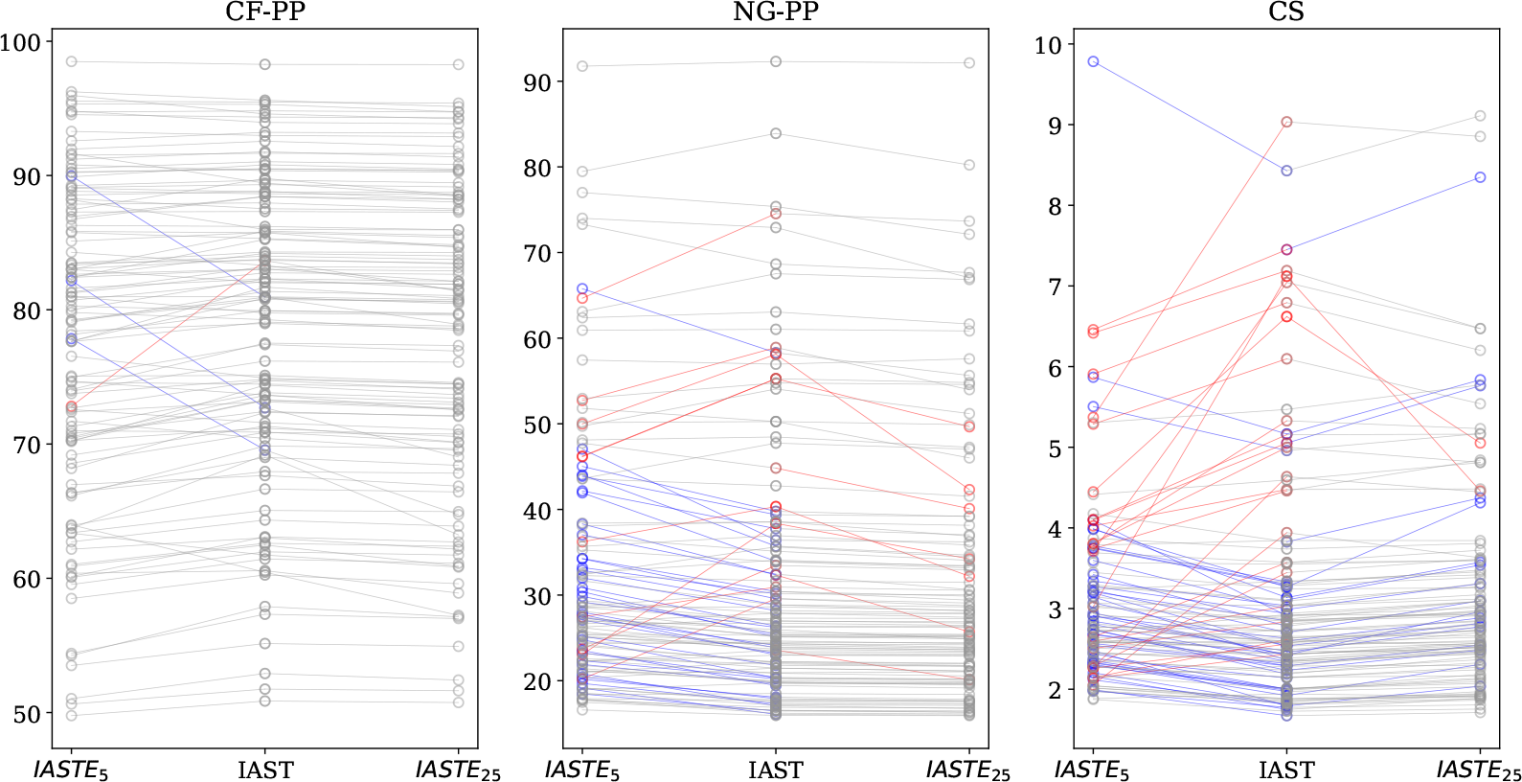
Purity (%) of output streams for a coal-fired power plant (CF-PP), a natural-gas-fired power plant (NG-PP), and confined spaces (CS) using only isotherm data at pressures above 5 and 25 mbar, IASTE_5_ and IASTE_25_, respectively, and IAST using all data. See also the caption to Figure 8.

An interesting question is whether IAST has similar difficulties if we remove the data at very low pressure. In Figure 11, we make a similar comparison for IAST with computational data removed below 5 mbar and below 25 mbar. In this comparison, one has to realize that we now have IAST predictions on both sides. Hence we are only comparing the effect of omitting the low-pressure data. In such a comparison for DSL, we have a convoluted effect as we see the differences between IAST and DSL and, on top of this, the effect of omitting low-pressure data. Yet, the effect is surprisingly small, and even for the confined spaces case one can obtain a decent ranking. If one uses DSL, one needs to fit the coefficients. Depending on the fitting procedure and set of data, one may get different sets of parameters that optimally fit the data but may give very different results when extrapolating to low pressures. This is also illustrated in Figure 10. For coal, we see that the ranking of DSL does not depend on the fitting, while for natural gas and confined spaces, we get a completely different ranking. The IAST procedure does not suffer from this and therefore yields a more reliable extrapolation to low pressures.

## Conclusion

In this study, we developed a robust workflow that optimally computes pure component isotherms at a given temperature and for a given guest molecule. The workflow was developed and tested for a set of 50 diverse MOF structures and later on applied to 500 other structures.

We showed that IAST can accurately predict binary uptake for a mixture of CO_2_ and N_2_, provided that the pure component isotherms are known up to sufficiently low and high pressures. We highlighted the differences between the use of IAST and extended DSL and were able to prove that IAST as a numerical tool is more robust and accurate than analytical methods, in this case DSL. Moreover, our method does not rely on fitting isotherm data to a particular model. This is important for screening a large number of materials where it is nearly impossible to manually inspect the quality of the fit to the data.

We observed that the performance ranking of materials, given a process design, is not only sensitive to the quality of the pure component isotherms but also to the selected thermodynamic description of the binary uptake data. The ranking of materials for a given key process performance indicator depends on whether we use IAST or DSL.

From a process simulation point of view, predicting mixture isotherms using IAST requires more CPU time than using a method that provides the mixture isotherm in an analytical form (e.g., DSL). This can be problematic, i.e., very computationally expensive, if one needs to optimize the process. Possibilities to address the CPU issue include fitting the IAST mixture predictions to B-splines as shown in the work of Santos et al.,^50^ or precomputing the mixture isotherms on a grid and using interpolation techniques.^51^

## Data Availability

All the details of the simulations (input files, structures, pure component isotherms, and mixture simulations) can be found on the Materials Cloud^39^ at https://archive.materialscloud.org/record/2023.68.

## Supporting Information Available

Details of the accuracy of the Clausius–Clapeyron relations, dual site Langmuir fitting, and process modeling (PDF)
