# Supplementary material for: A Robust Framework for Generating Adsorption Isotherms to Screen Materials for Carbon Capture
Source: Ind Eng Chem Res. 2023 Jun 20;62(26):10252–65. doi: 10.1021/acs.iecr.3c01358 (PMC10326871; doi:10.1021/acs.iecr.3c01358)
Supplement: Supplementary file 1 — ie3c01358_si_001.pdf [file ie3c01358_si_001.pdf]

Supporting Information: A Robust Framework for Generating Adsorption Isotherms to Screen Materials for Carbon Capture

Elias Moubarak,[†] Seyed Mohamad Moosavi,[†,‡] Charithea Charalambous,[¶] Susana Garcia,[¶] and Berend Smit[*,†]

†*Laboratory of Molecular Simulation, Institut des Sciences et Ingénierie Chimiques, École Polytechnique Fédérale de Lausanne (EPFL), Rue de l'Industrie 17, CH-1951 Sion, Valais, Switzerland*

‡*Department of Chemical Engineering & Applied Chemistry, University of Toronto, Ontario M5S 3E5, Canada*

¶*The Research Centre for Carbon Solutions (RCCS), School of Engineering and Physical Sciences, Heriot-Watt University, EH14 4AS Edinburgh, United Kingdom*

E-mail: berend.smit@epfl.ch

Abstract

The supporting information gives details on the accuracy of the Clausius-Clapeyron relations, the details of the fitting of the DSL model, and the process modelling main assumptions.

Contents

1 Clausius-Clapeyron Relation **S-3**

2 Dual-Site Langmuir Fitting **S-8**

3 Process Modeling **S-15**

3.1 Materials Ranking S-16

References **S-18**

1 Clausius-Clapeyron Relation

The Clausius-Clapeyron relation is used in this study to predict pure component isotherms at different temperatures given an isotherm at a reference temperature. Figure S1 helps understand how the relation is applied in practice.

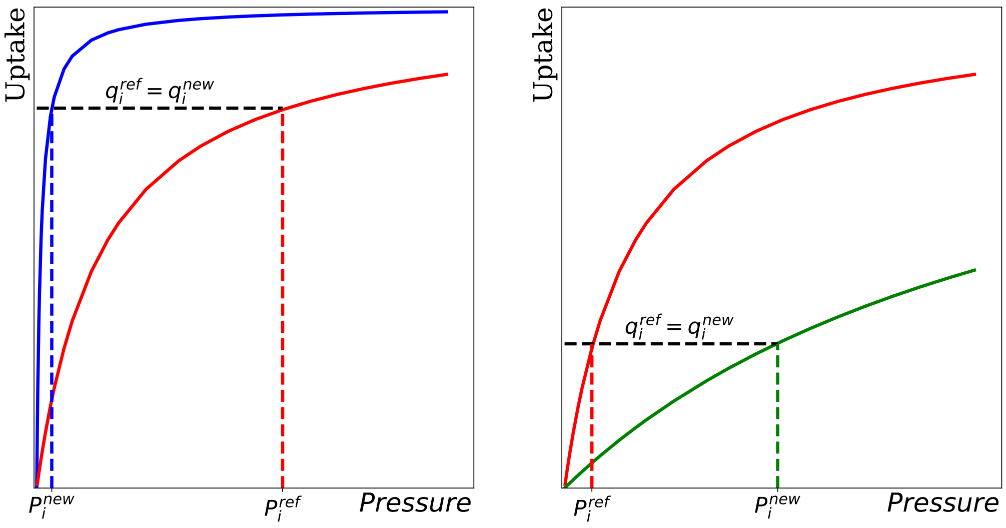

Figure S1: The reference isotherm at T_{ref} is in red. On the left, the predicted isotherm in blue is at $T_{\text{new}} < T_{\text{ref}}$. On the right, the predicted isotherm in green is at $T_{\text{new}} > T_{\text{ref}}$

The workflow developed to compute pure component isotherms ensures that the reference CO_2 and N_2 isotherms are thermodynamically consistent, i.e., the uptake is a monotonically increasing function of pressure:

$$\frac{q_{i+1}^{ref} - q_i^{ref}}{P_{i+1}^{ref} - P_i^{ref}} \geq 0 \tag{S1}$$

Where the numerator and denominator are both positive.

Consequently, the extrapolated isotherms, using the Clausius-Clapeyron relation, must also be thermodynamically consistent, as they are derived from the reference isotherms. Given that Clausius-Clapeyron shifts the pressure values, keeping the uptake constant, it follows from equation S1 that, for extrapolated isotherms to be monotonically increasing,

the relation described in equation S2 must hold:

$$P_{i+1}^{new} - P_i^{new} \geq 0 \tag{S2}$$

Substituting equation 13 into equation S2 and rearranging terms gives the following relation:

$$\frac{P_{i+1}^{ref}}{P_i^{ref}} > \exp\left(\frac{\Delta H_{\mathrm{ads_i}}^{ref} - \Delta H_{\mathrm{ads_{i+1}}}^{ref}}{R}\left(\frac{1}{T_{new}} - \frac{1}{T_{ref}}\right)\right) \tag{S3}$$

Equation S3 shows that the ratio of two consecutive pressure points P_i^{ref} and P_{i+1}^{ref} is related to the difference of their corresponding isosteric heats of adsorption, $\Delta H_{\mathrm{ads_i}}^{ref}$ and $\Delta H_{\mathrm{ads_{i+1}}}^{ref}$, respectively.

In the grand canonical ensemble, the isosteric heat of adsorption is calculated from fluctuations in the energy of the system and the number of particles.[S1] Therefore, at higher density, i.e., higher isotherm pressure, very long simulations are required to accurately calculate the isosteric heat of adsorption. Therefore, the isosteric heat of adsorption data is computed with high uncertainties.

The noisy nature of the isosteric heat data makes it almost impossible to meet the criterion described in equation S3. A trivial solution would be to use the average value of the isosteric heat of adsorption. However, reducing the data to a single value fails to include the effect of density on the heat of adsorption. In an attempt to reduce the noise in the data, we implemented fitting (linear regression and cubic spline approximation) and smoothing techniques (cumulative and exponential moving averages). They all failed to produce thermodynamically consistent isotherms when extrapolating to different temperatures. Therefore, we implemented an exponential filtering technique to reduce the noise in the data and meet the criterion. The technique can be summarized in the following relation:

$$\Delta H_{\mathrm{ads_i}}^{ref} = \frac{\sum_{j=0}^{i} \Delta H_{\mathrm{ads_j}}^{ref} \exp\left(-\alpha \frac{P_i}{P_j}\right) + \sum_{k=i}^{N} \Delta H_{\mathrm{ads_k}}^{ref} \exp\left(-\alpha \frac{P_k}{P_i}\right)}{\sum_{j=0}^{i} \exp\left(-\alpha \frac{P_i}{P_j}\right) + \sum_{k=i}^{N} \exp\left(-\alpha \frac{P_k}{P_i}\right)} \tag{S4}$$

Where α is the specific damping factor that is unique to each structure, an optimum value of α exists for each temperature. The lower the value of α, the higher the range of temperature that can be covered by the Clausius-Clapeyron relation. Therefore, α was computed in such a way to include the temperatures of interest up to 125 °C.

In this study, the Clausius-Clapeyron relation is used to predict the pure component isotherms for CO_2 and N_2 at 50, 75, 100, and 125 °C for each of the 50 sub-selected structures given a reference temperature of 25 °C. Then, the extrapolated isotherms were used to compute the binary adsorption data for a range of CO_2 partial pressures (0.0004 to 0.95) using IAST. These calculations were compared to the values obtained given the pure component isotherms generated using the workflow at the same temperatures. Figure S2 and Figure S3 show the error profiles for CO_2 and N_2 respectively. The percent errors were obtained using the following relation:

$$\text{Percent Error} = \frac{100|q_{i,\text{extrapolated}} - q_{i,\text{workflow}}|}{q_{i,\text{workflow}}} \tag{S5}$$

Where i can be either CO_2 or N_2

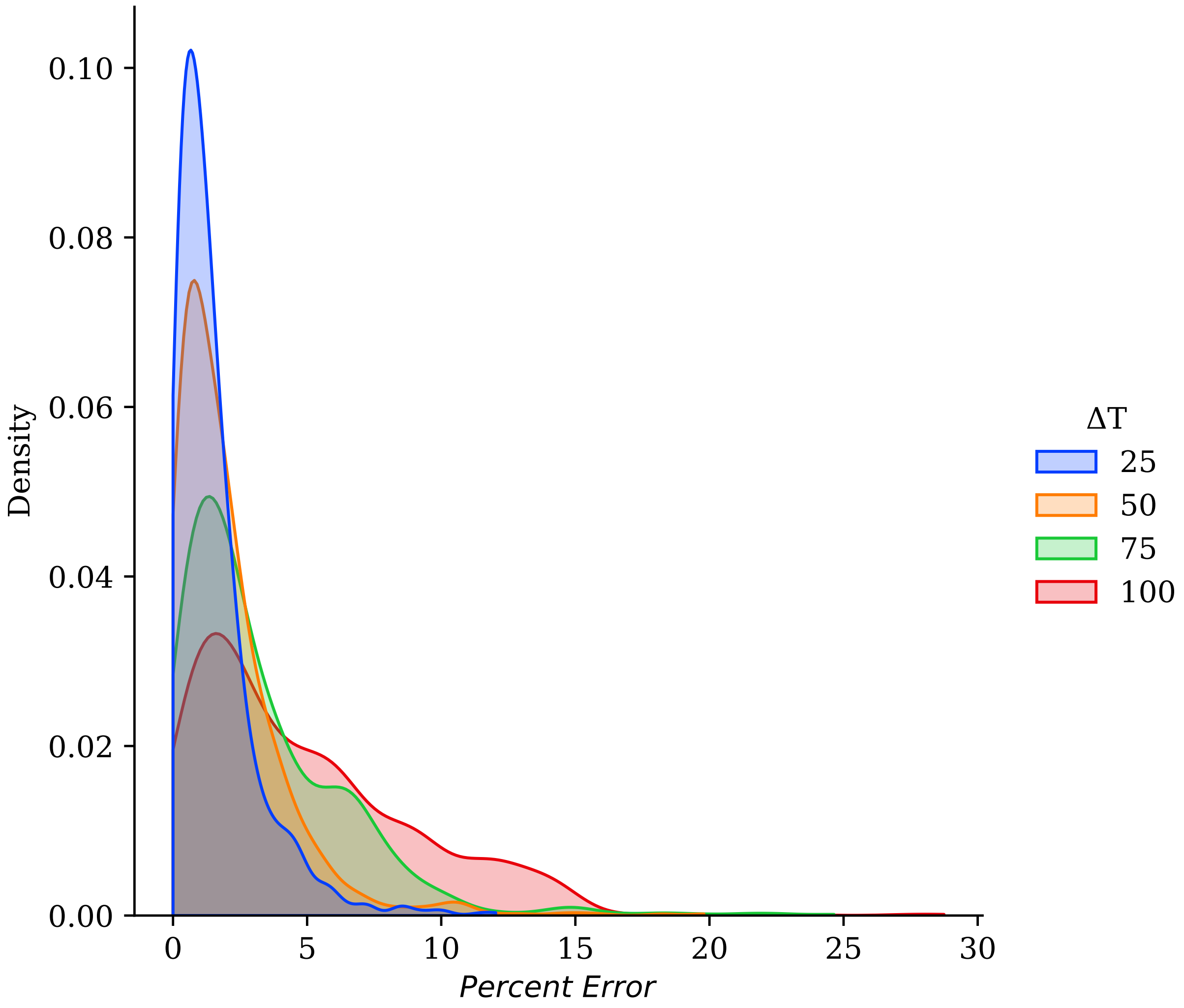

Figure S2: Percent error distribution of binary CO_2 uptake at a temperature difference (ΔT) of 25, 50, 75, and 100 for the subset of 50 structures, given the reference isotherm at 25 °C.

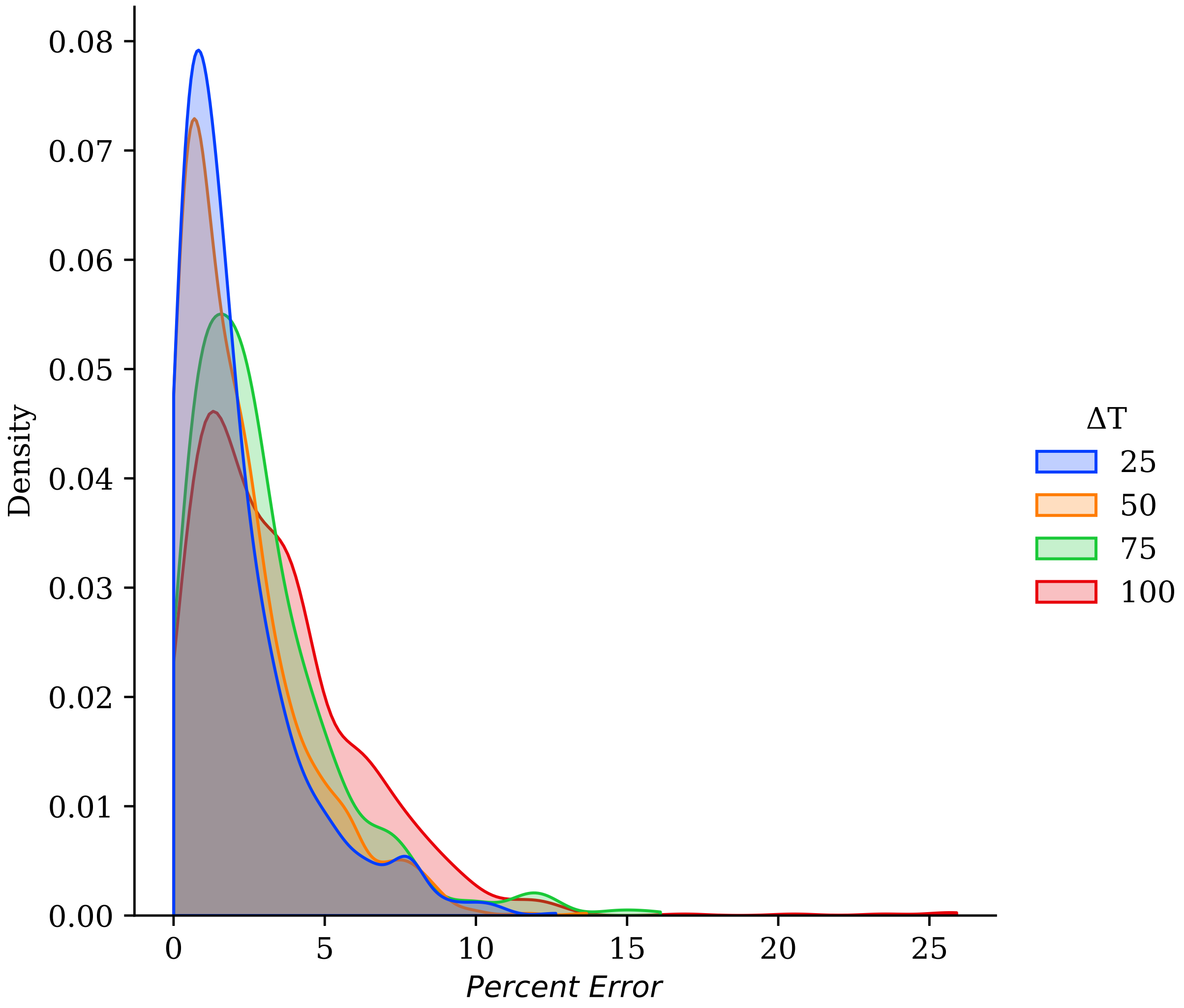

Figure S3: Percent error distribution of binary N_2 uptake at a temperature difference (ΔT) of 25, 50, 75, and 100 for the subset of 50 structures, given the reference isotherm at 25 °C.

In figures S2 and S3, the percent error distribution of the predicted binary CO_2 and N_2 uptakes, respectively, for all temperature differences display a peak between 1% and 3%. This shows that using Clausius-Clapeyron to extrapolate to different temperatures is accurate enough for most of the binary CO_2 and N_2 predictions. However, as the temperature difference increases, the error distribution flattens, and higher errors are attained. For example, in the extreme case of 125 °C, very few predictions are calculated with 25% error. One way to overcome this issue is to use another reference isotherm at a relatively higher temperature, 75 °C for example, to minimize the temperature difference when extrapolating

at high temperatures.

2 Dual-Site Langmuir Fitting

In the following section, we discuss the shortcomings of DSL fitting affecting mainly the fits of pure component CO_2 isotherms, given that CO_2 is the strongly adsorbed component. Also, we show that using IAST to predict binary adsorption data is a more reliable tool than extended DSL for the full set of structures.

For instance, a different set of model parameters can be obtained by using different initial guesses. Figure S4 shows, for four structures, that in the high-pressure region, where the majority of data points lie, the different sets of parameters give similar fits. However, in the vicinity of the Henry regime, different initial guesses give very different predictions. It is also evident from figure S4 that for a given set of initial guesses corresponding to the solid lines, DSL fits describe better the pure component data. Those "educated guesses" follow some simple rules: (1) the saturation loadings, q_{sat1} and q_{sat2} in equation 16, are estimated using the highest uptake value for CO_2 and (2) the b_1 and b_2 parameters in equation 16, are estimated from the Langmuir model using the Henry coefficients ($K_H = b * q$).

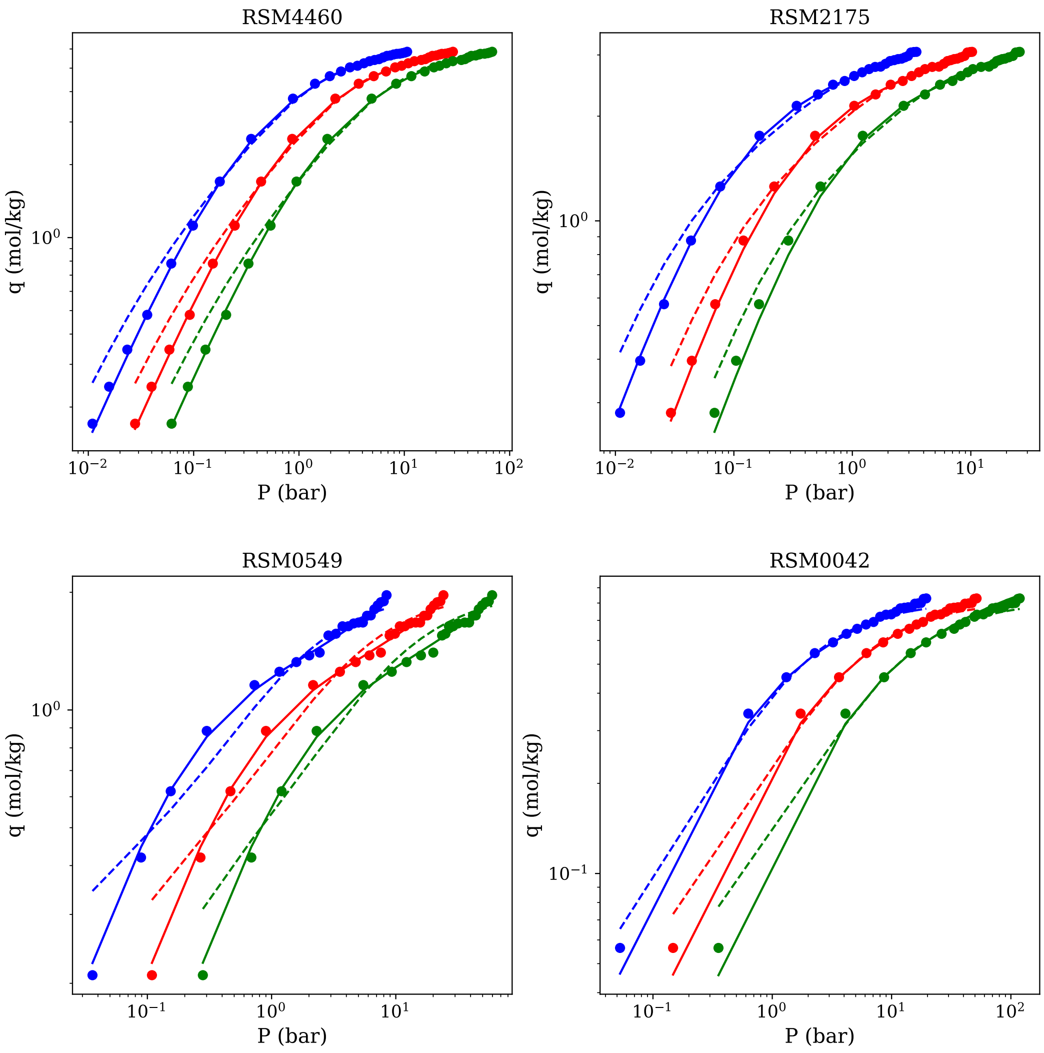

Figure S4: DSL fits for four different structures at 25, 50 and 75 °C, corresponding to blue, red, and green, respectively. Data points, dotted lines, and solid lines correspond to pure component data, DSL fits using random guesses, and DSL fits using "educated guesses," respectively.

In addition, fitting can be done either by putting more weight on data points at low pressure to force the correct Henry coefficient or by considering all data points to have the same weight. Figure S5) shows that forcing the correct Henry coefficient affects the accuracy of the fits at high pressure. It also shows that the accuracy of the fits at low pressure is

compromised when we consider all data points to have the same weight.

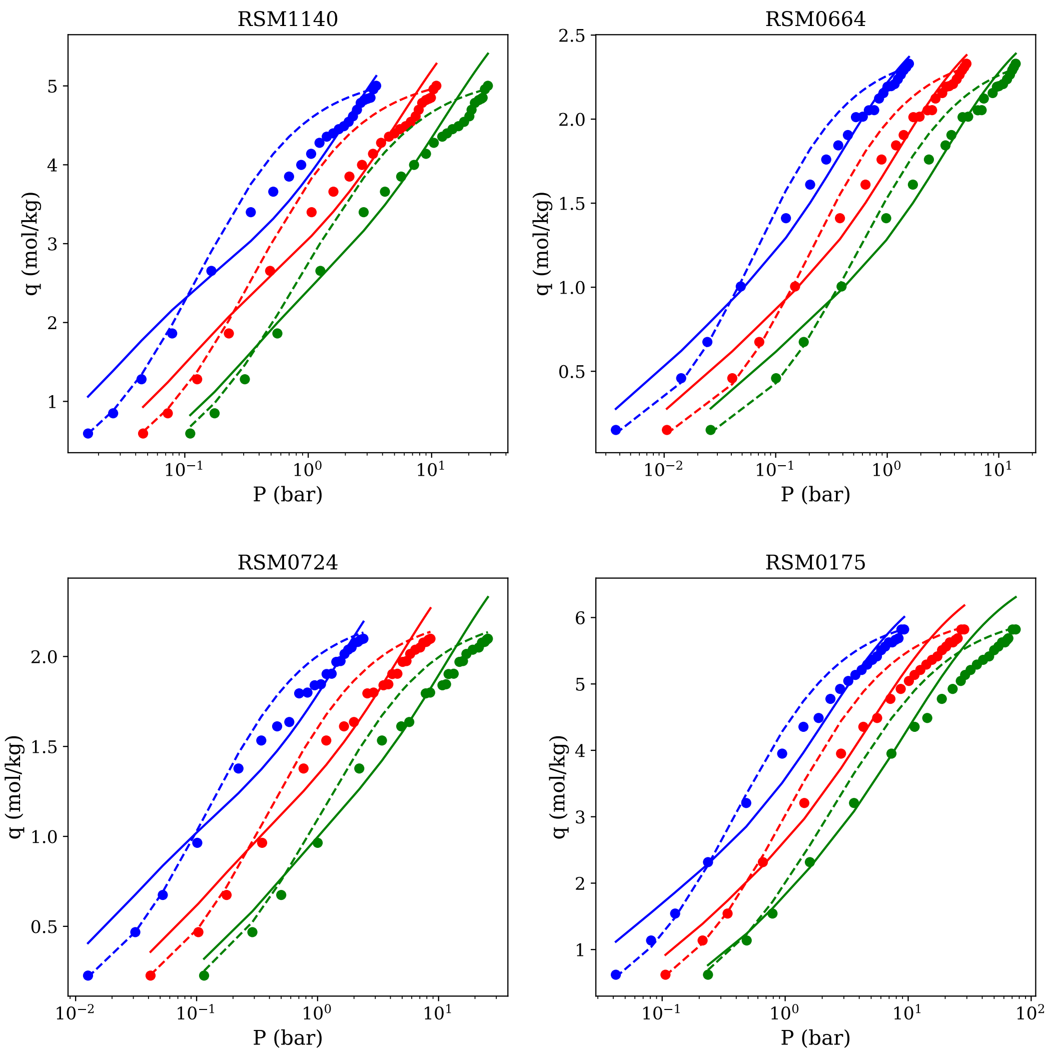

Figure S5: DSL fits for four different structures at 25, 50, and 75 °C, corresponding to blue, red, and green, respectively. Data points, dotted lines, and solid lines correspond to pure component data, DSL fits where more weight was put on low-pressure points, and DSL fits where all points have the same weight, respectively.

Furthermore, figure S6 shows that DSL always over-predicts saturation uptake compared to the value given by the pure component data, and differences are highest for high saturation pressures.

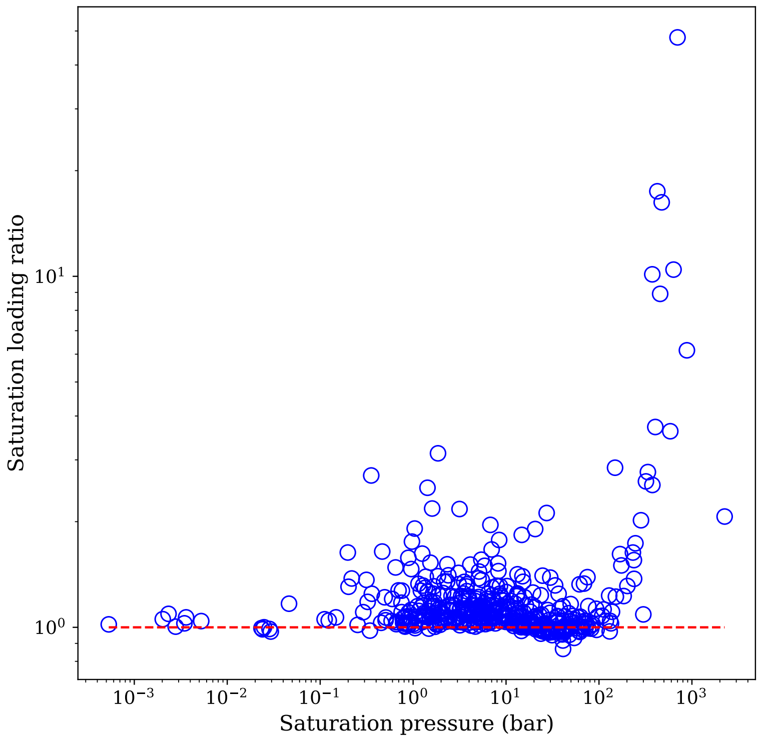

Figure S6: Saturation loading ratio as a function of saturation pressure. The ratio is computed by dividing the saturation uptake obtained from fitting the data by the saturation uptake given the pure component isotherms

Moreover, the fitting procedure might give a set of physically unacceptable parameters, such as positive heat of adsorption values for both CO_2 and N_2, or saturation uptakes in the range of thousands. Yet, as shown in figure S7), these parameters might still lead to accurate fits mathematically. Nevertheless, those fits do not abide by the thermodynamics of the DSL model. For example, positive heats of adsorption are obtained, which renders adsorption an endothermic process.

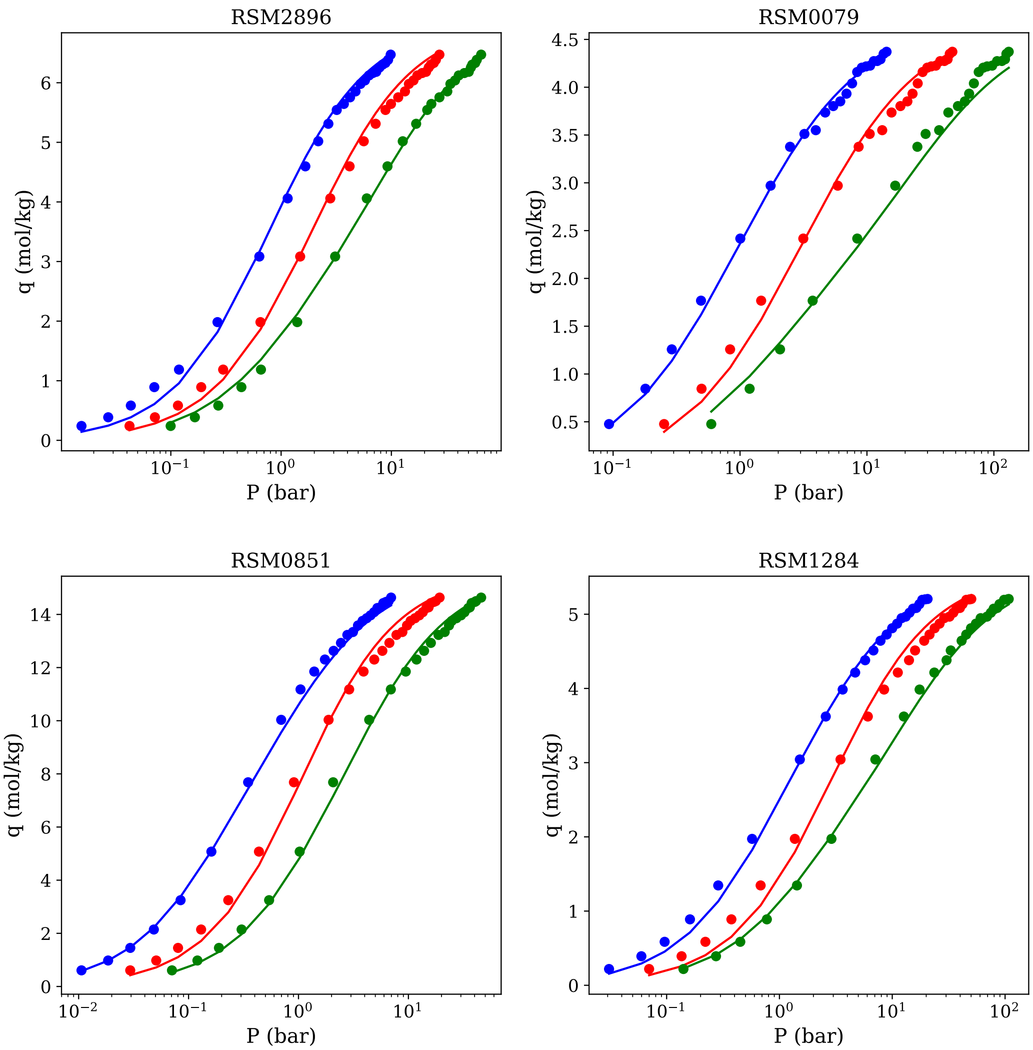

Figure S7: DSL fits for four different structures at 25, 50, and 75 °C, corresponding to blue, red, and green, respectively. Data points and solid lines correspond to pure component data and DSL fits.

Finally, we predicted binary adsorption data for CO_2 and N_2 using IAST and extended DSL with and without forcing the correct Henry coefficient, given the structures for which the fitting went well (400 out of 500). Those predictions were compared to binary GCMC adsorption data at the adsorption conditions of temperature and CO_2 partial pressure for

three different case studies: coal-fired power plant (CF-PP), natural gas-fired power plant (NG-PP), and confined spaces (CS). Refer to table S1 for the gas composition and adsorption temperature. Figure S8 shows that for the three cases and both guest molecules, IAST predictions are more reliable and accurate than using extended DSL.

(a)

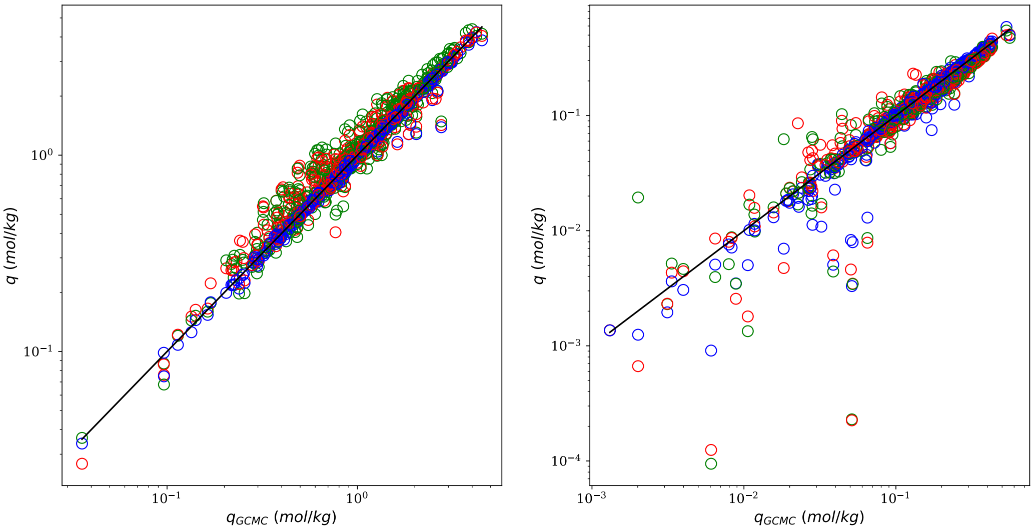

(b)

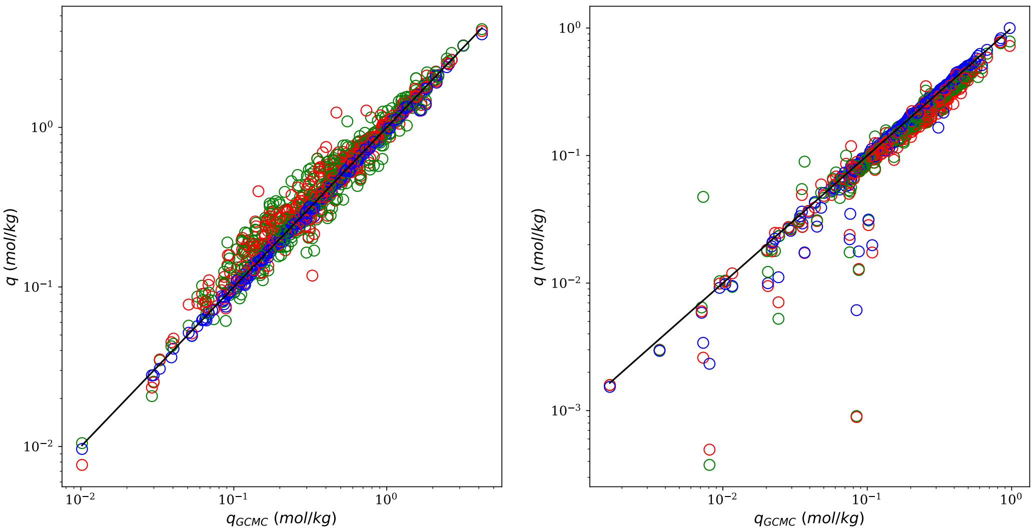

(c)

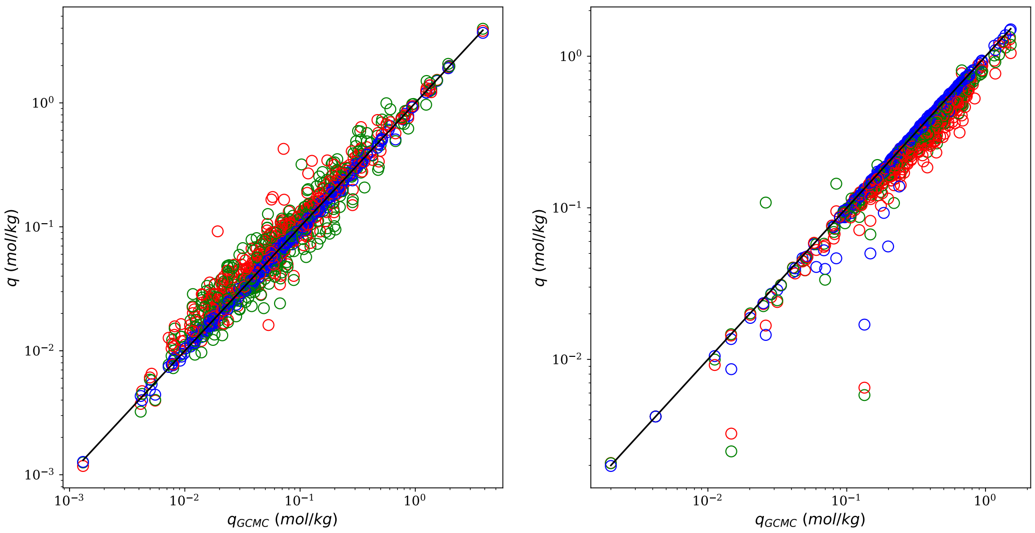

Figure S8: CO_2 (left) and N_2 (right) binary adsorption using IAST (blue) and extended DSL with and without forcing the correct Henry coefficient (green and red respectively) for coal-fired power plant (a), natural gas-fired power plant (b), and confined spaces (c).

3 Process Modeling

The TSA model implemented in this study is built in python around some basic assumptions:

- The inlet stream is a dry binary mixture of CO_2 and N_2 and is considered as an ideal gas. It also goes without saying that both components are adsorbable.
- Equilibrium between the gas and adsorbed phases is reached instantaneously because mass transfer resistance is negligible.
- Heating and cooling are assumed to be carried out with heating fluids flowing along the column with a homogeneous temperature distribution. Also, the heat transfer resistance is negligible.
- The radial gradients, thermal dispersion, and axial mixing are negligible.
- Pressure drop across the bed is negligible.

In this work, we consider capturing CO_2 from three different sources at ambient pressure (1.01325 bar): (1) a coal-fired power plant (CF-PP), (2) a natural gas power plant (NG-PP), and (3) confined spaces (CS). The product is collected at the desorption temperature of 125°C. Table S1 summarizes the process parameters.

Using the TSA process, one can rank materials based on different key performance indicators ($KPIs$) such as purity, working capacity (WC), and thermal energy ($E_{thermal}$) requirements. Those KPIs are defined as:

$$Purity = \frac{N_{\mathrm{CO_2,product}}}{N_{\mathrm{product}}} \tag{S6}$$

$$WC = \frac{N_{\mathrm{CO_2,product}}}{m_{\mathrm{s}}} \tag{S7}$$

Here $N_{\mathrm{CO_2},product}$ and N_{product} are the amount of CO_2 and the total amount leaving the column in step (ii) (refer to figure 7), respectively and m_{s} is the mass of the solid.

Table S1: Process parameters describing the inlet flue gas and bed characteristics.

Case Study	Inlet CO_2 fraction (mol basis)	$T_{adsorption}$ (°C)
CF-PP	0.1521[S2]	37
NG-PP	0.0432[S2]	37
CS	0.0040[S3]	25

Parameter	Value	Unit
CO_2 heat capacity (c_p)	38.51	$J\,mol^{-1}\,K^{-1}$
CO_2 heat capacity (c_v)	30.30	$J\,mol^{-1}\,K^{-1}$
N_2 heat capacity (c_p)	29.16	$J\,mol^{-1}\,K^{-1}$
N_2 heat capacity (c_v)	20.85	$J\,mol^{-1}\,K^{-1}$
Bed porosity	0.37[S4]	–
Pellet porosity	0.35	–

$$E_{\text{thermal}} = \Delta H_{\text{sens}} + \Delta H_{\text{ads}} \tag{S8a}$$

$$\Delta H^j_{\text{sens}} = m_{\text{s}}\ c_{\text{p,s}}\ (T_j - T_{(j-1)}) + \left(c_{\text{p,CO}_2} N^j_{\text{CO}_2\text{,col}} + c_{\text{p,N}_2} N^j_{\text{N}_2\text{,col}}\right)(T_j - T_{(j-1)}) \tag{S8b}$$

$$\Delta H^j_{\text{ads}} = m_{\text{s}}\left(\Delta H_{\text{CO}_2}\Delta N^j_{\text{CO}_2\text{,ads}} + \Delta H_{\text{N}_2}\Delta N^j_{\text{N}_2\text{,ads}}\right) \tag{S8c}$$

Here, ΔH_{sens} and ΔH_{ads} are the process total sensible heat and heat of adsorption ($kJ\,mol^{-1}$) requirements per TSA cycle. ΔH^j_{sens} and ΔH^j_{ads} are the sensible heat and heat of adsorption at step j. T is the temperature of the column, c_p denotes the specific heat capacity, N_i denotes the amount of species i, ΔH_i refers to the heat of adsorption of species i, and ΔN_i is the difference in the amount of species i between step j and step $(j-1)$. The subscripts col, ads, and s refer to the column, the adsorbed phase, and the solid, respectively.

3.1 Materials Ranking

In figure 8, we showed the materials ranking for the three case studies based on the purity of the CO_2 rich product stream. In figures S9 and S10, we show the materials ranking using two different KPIs, working capacity ($mol\,kg^{-1}$) and specific heat requirements ($MJ\,mol^{-1}$) respectively.

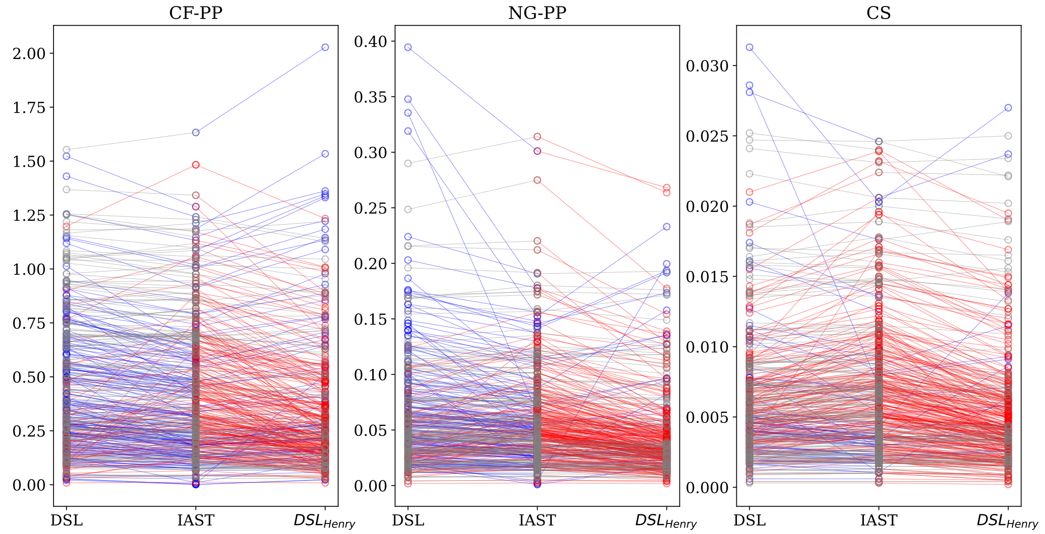

Figure S9: Working capacity ($\mathrm{mol\,kg^{-1}}$) of the output stream for a coal-fired power plant (CF-PP), natural gas-fired power plant (NG-PP), and confined spaces (CS). In these figures we compare IAST with DSL and DSL_{Henry}, see also the caption to figure 8

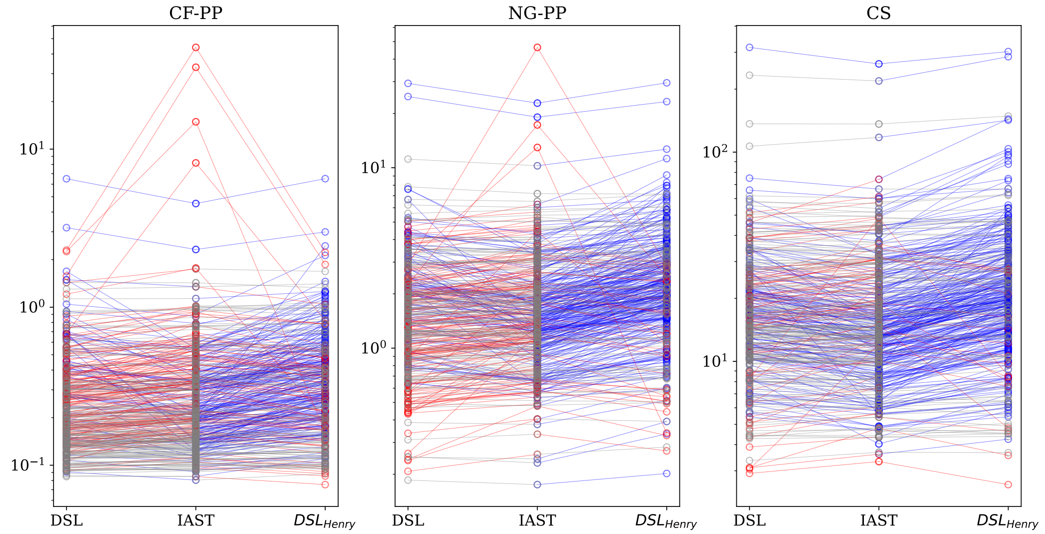

Figure S10: Specific heat requirements ($\mathrm{MJ\,mol^{-1}}$) of the output stream for a coal-fired power plant (CF-PP), natural gas-fired power plant (NG-PP), and confined spaces (CS). In these figures we compare IAST with DSL and DSL_{Henry}, see also the caption to figure 8

References

(S1) Vlugt, T.; García-Pérez, E.; Dubbeldam, D.; Ban, S.; Calero, S. Computing the Heat of Adsorption Using Molecular Simulations: the Effect of Strong Coulombic Interactions. *Journal of chemical theory and computation* **2008**, *4*, 1107–1118.

(S2) Fernandez, E. S.; Goetheer, E. L.; Manzolini, G.; Macchi, E.; Rezvani, S.; Vlugt, T. J. Thermodynamic Assessment of Amine Based CO_2 Capture Technologies in Power Plants Based on European Benchmarking Task Force Methodology. *Fuel* **2014**, *129*, 318–329.

(S3) Perry, J. L.; Sargusingh, M. J.; Toomarian, N. *AIAA SPACE 2016*; 2016; p 5461.

(S4) Chhabra, R.; Richardson, J. Particulate Systems. *Non-Newtonian flow in the process industries.(Elsevier, Amsterdam, 1999)* **2008**, 206–259.
